# CRISPR/Cas9-Mediated Metabolic Engineering of Endophytic *Pseudomonas loganensis* sp. nov. for the Production of Nutritionally Valuable Carotenoids

**DOI:** 10.1021/acsomega.5c05877

**Published:** 2026-01-02

**Authors:** Nuriye Arslansoy, Melisa Zulal Karaman, Ozkan Fidan

**Affiliations:** † Department of Bioengineering, Graduate School of Science and Engineering, 346448 Abdullah Gül University, Kayseri 38080, Turkey; ‡ Department of Bioengineering, Faculty of Natural and Life Sciences, 346448 Abdullah Gül University, Kayseri 38080, Turkey

## Abstract

Carotenoids with significant nutritional and antioxidant properties have been widely utilized in the food, feed, pharmaceutical, and cosmetic industries. They improve the nutritional value of foodstuffs and have been used as natural food colorants. However, their current supply chain is mainly dependent on extraction from plants and chemical synthesis, both of which have bottlenecks, including environmental concerns, toxicity, and allergenicity. To address global demand for sustainable and environmentally friendly production of nutrients, we engineered the endophytic *Pseudomonas loganensis* sp. nov. as a niche microbial chassis for nutritionally valuable carotenoid production. Using CRISPR-Cas9, we knocked out key carotenogenic genes to construct strains capable of producing zeaxanthin, lycopene, and β-carotene. Additionally, an overexpression plasmid was introduced to produce astaxanthin. HPLC analysis confirmed the successful production of four target carotenoids. The culture conditions and media compositions were optimized using response surface methodology, resulting in a ∼5-fold increase in the titers of zeaxanthin (13.4 mg/L), lycopene (9.67 mg/L), and β-carotene (23.53 mg/L), and a ∼12-fold increase in astaxanthin titer (1 mg/L) compared to LB medium without optimization. Our results indicate the potential of endophytic bacteria as a microbial chassis for carotenoid bioproduction, underscoring the potential of synthetic biology to contribute to global efforts toward nutritional security and sustainable food systems.

## Graphical abstract

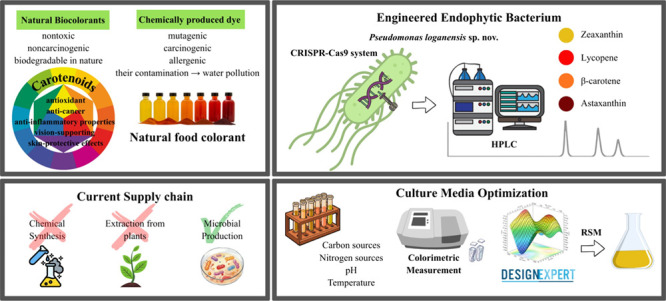

## 1 Introduction

Carotenoids are pigment molecules and a class of terpenoids composed of isoprenoid (C5) units. They are predominantly found in photosynthetic organisms such as plants, algae, fungi, and some bacterial species. They are classified based on their carbon numbers, including C30, C40, and C50, with C40 carotenoids being the most extensively studied group due to their high abundance in nature. Based on the functional groups in their structures, carotenoids can be categorized as carotenes and xanthophylls. Carotenes only have hydrocarbon structures (e.g., β-carotene, lycopene), while xanthophylls contain oxygen which affects their solubility, polarity, and biological activity. Xanthophylls can be further categorized based on their functional groups, including hydroxy, epoxy, and ketocarotenoids.^1^

Carotenoids play essential roles in human health, mainly due to their strong antioxidant properties.^2^ In addition to antioxidant activity, they were reported to exhibit antiviral, antidiabetic, anticancer, and pro-vitamin A activities.^3^ Additionally, carotenoids have proven health benefits such as increasing cognitive abilities, reducing the risk of chronic diseases, preventing age-related eye disorders, and therefore, have gained attention in the pharmaceutical industry.^3,4^ For instance, β-carotene serves as a precursor to vitamin A, which has a crucial role in eye functioning.^5,6^ Astaxanthin has been suggested to enhance treatment efficacy for COVID-19 by suppressing the cytokine storm.^7^ Since animals and humans cannot synthesize carotenoids, it is important to take them through diet or supplementation. Hence, their use as food ingredients is beneficial for human health. Furthermore, carotenoid-biofortified feed and food might offer a promising strategy to combat vitamin A deficiency.^8,9^ Therefore, they are commonly used as natural colorants for food coloration, egg yolk, and aquatic organism pigmentation.^10^ All of these benefits provide a wide range of uses in various industries and increase the demand for carotenoids. Carotenoids have a huge market size and expansion of the market size due to increased demand is expected from USD 1.5 billion in 2017 to USD 2 billion by 2026.^11^

The current supply chain for carotenoids is mainly dependent on extraction from plants and chemical synthesis. However, these strategies have some disadvantages and bottlenecks.^12^ Use of organic solvents for both the chemical synthesis and plant extraction poses environmental concerns, while factors such as climate variability and seasonal changes affect plant availability. In addition, cultivating carotenoid-rich plants is costly and time-consuming. Since the synthetic carotenoids produced by chemical synthesis might lead to health problems such as toxicity and increased allergenicity. This has contributed to a growing interest in microbial production as an alternative.^11^ Microbial production can overcome these disadvantages and bottlenecks and offers a more sustainable and efficient approach. Indeed, microbial production is essential for meeting industrial demand due to its scalability, efficiency, and lower cost.^13^ Currently, microalgae, bacteria, and fungi are commonly used as microbial carotenoid producers.^14,15^ Furthermore, metabolic engineering strategies have facilitated carotenoid biosynthesis in non-native microbial hosts.^4^ Furthermore, genetic engineering techniques have been employed to enhance production of carotenoids.^16–18^

Given the pressing need for sustainable nutrient sources to support a growing population, microbial production platforms present a sustainable and environmentally friendly solution.^19,20^ Synthetic biology enables the development of such platforms for producing high-value nutrients such as carotenoids, which are essential for vision, immune function, and antioxidant defense.^21,22^ Particularly, CRISPR-mediated metabolic engineering can significantly contribute to global food security by enabling the precision fermentation of nutritionally valuable compounds, including carotenoids.^23,24^ Genetically engineered microorganisms support sustainable production platforms that can operate independently of agricultural limitations such as climate instability, land scarcity, and fluctuating crop yields.^24^ This work aimed to contribute to the global effort toward achieving United Nations Sustainable Development Goal 2: Zero Hunger, by providing sustainable, environmentally benign, and nonplant-based food ingredient production.

Plant-associated microorganisms, known as endophytes, are viable sources of natural products. They are capable of synthesizing primary and secondary metabolites with diverse biological activities that influence the host metabolism. As a result, endophytes serve as rich reservoirs of novel bioactive compounds. Moreover, modern genetic tools enable the modification of endophytes to harness their potential for producing various antimicrobial, antiviral, and anticancer molecules.^25^ *Pseudomonas* sp. 102515 was originally isolated as an endophyte from the leaves of *Taxus chinensis*. In our recent study, this strain was biochemically characterized and its genome analysis revealed that it is a novel *Pseudomonas* species and renamed as *Pseudomonas loganensis* sp. nov.^26,27^ The isolate displays yellow pigmentation, which led to the discovery that it naturally produces zeaxanthin diglucoside. Characterization of its carotenoid biosynthetic gene cluster revealed that key intermediates such as lycopene, β-carotene, and zeaxanthin can be produced through the knockout of certain carotenoid genes (Figure 1).^28^ For instance, *crtY* is responsible for the cyclization of lycopene, leading to the biosynthesis of β-carotene. Therefore, the knockout of *crtY* results in the accumulation of lycopene in the engineered strains. In addition to being a native carotenoid producer with a functional biosynthetic gene cluster, *P. loganensis* sp. nov. can grow under relatively high salt concentrations, suggesting that its endophytic lifestyle may confer salt stress tolerance. The capability of salt stress tolerance can offer economic benefits as it allows for more efficient production under the high-osmolarity conditions during industrial fermentations.^29,30^ Additionally, its ability to synthesize trehalose from maltose has also been reported, and its nitrogen-fixing capacity further underscores its potential for agricultural applications. These characteristics collectively make *P. loganensis* sp. nov. an attractive niche microbial chassis.

**Figure 1:**
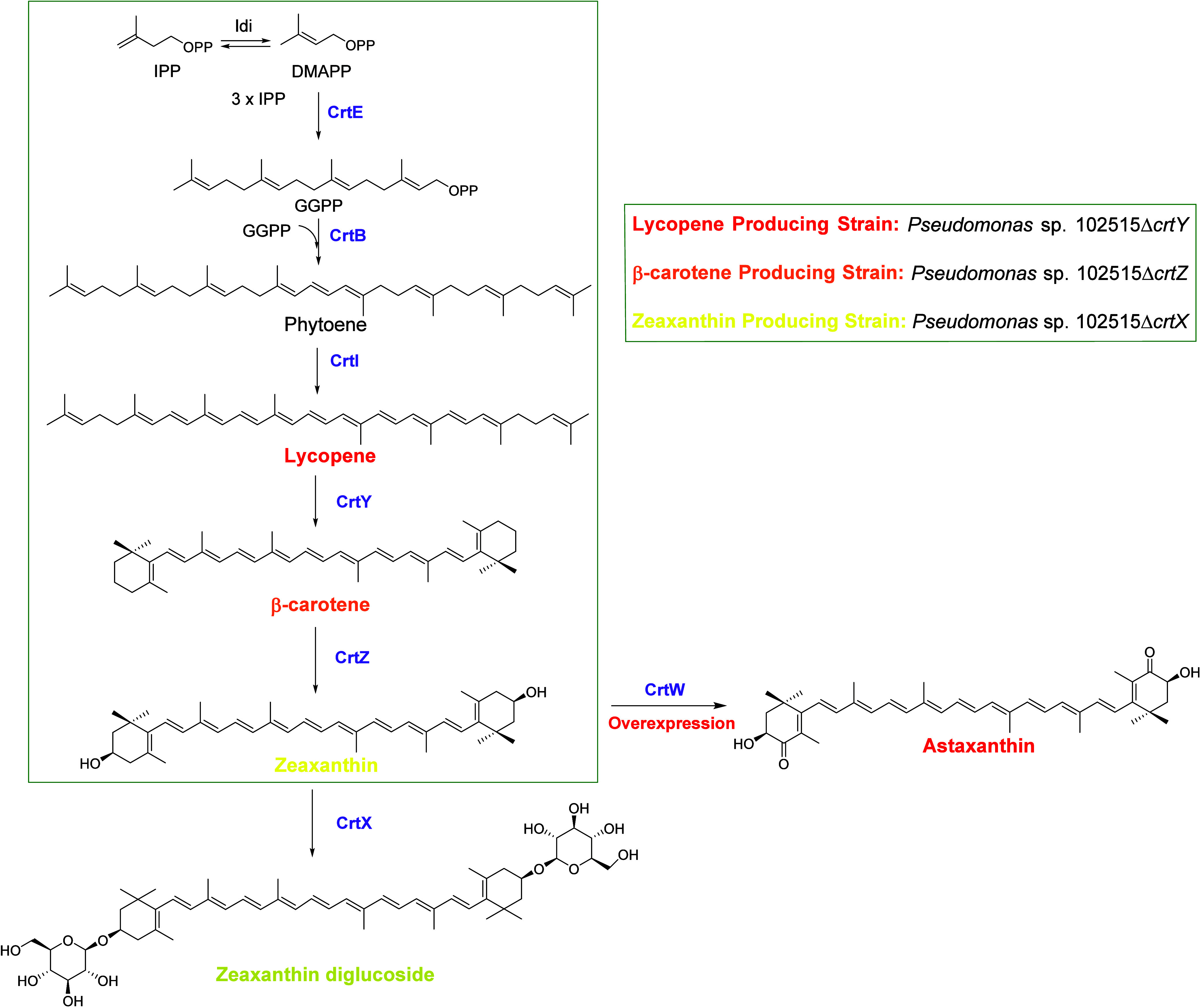
Carotenoid biosynthetic pathway in *P. loganensis* sp. nov. and engineered strains. The pathway for the *crtX* knockout strain is shown in the box. *crtW* is overexpressed using the *crtX* knockout strain to generate the astaxanthin-producing strain.

In this study, we engineered *P. loganensis* sp. nov., a native zeaxanthin diglucoside producer, to biosynthesize nutritionally and economically valuable carotenoids: zeaxanthin, lycopene, β-carotene, and astaxanthin.^28^ This was performed by knocking out zeaxanthin glucosyltransferase (*crtX*), lycopene β-cyclase (*crtY*), and β-carotene hydroxylase (*crtZ*) genes from the genome of *P. loganensis* sp. nov. using the CRISPR-Cas9 system to generate Δ*crtX*, Δ*crtY*, and Δ*crtZ* knockout strains, which produced zeaxanthin, lycopene, and β-carotene, respectively. Additionally, the β-carotene ketolase (*crtW*) gene from *Nostoc sphaeroides* PCC 7120 was cloned into an overexpression plasmid and transformed into the *crtX* knockout mutant to produce astaxanthin. The synthesized carotenoids from engineered strains were extracted and confirmed by TLC and HPLC analysis. To our knowledge, this is the first successful study establishing a biosynthetic platform for the production of four nutritionally significant carotenoids, zeaxanthin, lycopene, β-carotene, and astaxanthin, using an engineered *P. loganensis* sp. nov. The culture conditions and media compositions were optimized using response surface methodology to enhance the titers of each carotenoid in the engineered strains. This led to improving zeaxanthin, lycopene, and β-carotene titers by ∼5-fold and astaxanthin titer by ∼12-fold compared to the LB medium without optimization. Rather than positioning, *P. loganensis* sp. nov. as a direct competitor to established industrial strains, our results establish it as a genetically tractable and niche microbial chassis with unique biological features. Future metabolic engineering could be achieved through the development and application of CRISPR tools to this strain, including multiplex genome editing and promoter fine-tuning.

## 2 Materials and Methods

### 2.1 Strains, Media, and Plasmids

Strains and plasmids are shown in Table 1. Generally, *E. coli* Top10 cells were used for cloning experiments, and *P. loganensis* sp. nov.^28^ was used for genetic manipulations and carotenoid production. Luria–Bertani (LB) medium (Condalab) was utilized for bacterial growth in all steps, with 20 g/L of bacteriological agar (Condalab) and antibiotics added whenever required. LB medium was supplemented with kanamycin (50 μg/mL, Kan50 for *E. coli* and 30 μg/mL, Kan30 for *P. loganensis* sp. nov.), ampicillin (100 μg/mL, Amp100), gentamicin (35 μg/mL, Gen35), and tetracycline (10 μg/mL, Tet10 for *E. coli* and 25 μg/mL, Tet25 for *P. loganensis* sp. nov.). Culture conditions were maintained at 37 °C for *E. coli* Top10 and 28 °C for *P. loganensis* sp. nov., both at 220 rpm.

**Table 1:** Strains and Plasmids Used in This Study

strain or plasmid	description	source
strains		
*E. coli* Top10	F- mcrA Δ(mrr-hsdRMS-mcrBC) φ80 lacZΔM15Δ*l*acX74 recA1 araΔ139Δ(ara-leu)7697 galU galK rpsL (StrR) endA1 nupG	lab stock
*P. loganensis* sp. nov.	wild type, zeaxanthin diglucoside-producing strain	^28^
*P. loganensis* sp. nov. Δ*crtX*	zeaxanthin-producing knockout strain	this study
*P. loganensis* sp. nov. Δ*crtY*	lycopene-producing knockout strain	this study
*P. loganensis* sp. nov. Δc*rtZ*	β-carotene-producing knockout strain	this study
*P. loganensis* sp. nov. Δ*crtX*/pNAr18	astaxanthin-producing overexpression strain	this study
Plasmids		
pCas9	recombineering plasmid with constitutively expressed *cas9* and the araBAD promoter expressing αβγ	^31^
pJOE_pvdJ	*P. putida* KT2440 suicide plasmid with repair template for *pvdJ* knockout	^31^
pNAr7	*P. putida* KT2440 suicide plasmid with repair template for *crtX* knockout	this study
pNAr10	*P. putida* KT2440 suicide plasmid with repair template for *crtY* knockout	this study
pNAr14	*P. putida* KT2440 suicide plasmid with repair template for *crtZ* knockout	this study
pgRNAtet-IvaA	guide RNA plasmid targeting *lvaA*	^31^
pNAr8	guide RNA plasmid targeting *crtX*	this study
pNAr9	guide RNA plasmid targeting *crtY*	this study
pNAr15	guide RNA plasmid targeting *crtZ*	this study
pJET1.2	cloning vector	lab stock
pNAr11	blunt-ended β-carotene ketolase gene *crtW* cloned into pJET1.2	this study
pMiS1-ges-mva	*P. putida* KT2440 expression vector pMiS1 with geraniol synthase gene *ges* and 6 MVA pathway genes	^32^
pNAr17	*P. putida* KT2440 expression vector pMiS1 with *CaZEP* gene	this study
pNAr18	*P. putida* KT2440 expression vector pMiS1 with β-carotene ketolase gene *crtW*	this study

### 2.2 Plasmid Construction

PCR amplifications were performed using Phusion DNA Polymerase (ThermoFisher Scientific) with the primers listed in Table S1. Standard PCR reactions were conducted under standard PCR conditions. A modified protocol was developed for overlapping-extension (OE) PCR: A reaction mix containing 0.25 μM forward primer, 0.25 μM reverse primer, 1× buffer, 400 μM dNTPs, 3% DMSO, a variable amount of template, 0.02 U/μL Phusion DNA Polymerase, and dH_2_O up to 20 μL. The first 20 cycles were carried out without primers to allow fragment overlap, after which primers were added, and the PCR was continued for additional cycles under the same standard conditions.

#### 2.2.1 Construction of pJOE with Homologous Arms of the Target Genes

Genomic DNA of *P. loganensis* sp. nov. was isolated by a genomic DNA extraction kit (Transgen Biotech) and used as a template for the amplification of upstream and downstream homologous arms of the *crtX*, *crtY*, and *crtZ* genes by PCR. Amplified upstream and downstream homologous arms of the genes were purified from agarose gel using Gel Extraction Kit (Favorgen), hybridized, and amplified by OE PCR using upstream forward and downstream reverse primers. pJOE_pvdJ plasmid was digested with *Bam*HI and PmlI restriction enzymes (ThermoFisher Scientific). Digested pJOE_pvdJ and hybridized upstream-downstream homologous arms were purified from the gel and ligated by Gibson Assembly (TakaraBio In-Fusion Cloning). Constructed plasmids were verified through restriction digestion analysis, followed by confirmation via Sanger sequencing. The plasmid maps are given in Figure S1.

#### 2.2.2 Construction of pgRNAtet with N20 Sequence of the Target Genes

For the construction of pgRNAtet, the plasmid was amplified by PCR as two fragments, and the gene specific N20 PAM sequence was changed by primers. First, the pgRNAtet-F and pgRNAtet-R primer pair was used to obtain a 1.6 kb fragment. Then, pgRNAtet-CrtXYZ-R and pgRNAtet-CrtX-F primers were used to generate a 1.9 kb fragment with specific N20 sequence for *crtX* gene. These two PCR products were purified from an agarose gel and ligated by Gibson Assembly (TakaraBio In-Fusion Cloning). The same protocol was followed for *crtY* and *crtZ* genes using pgRNAtet-CrtY-F and pgRNAtet-CrtZ-F primers instead of pgRNAtet-CrtX-F primer. Constructed plasmids were verified through restriction digestion analysis, followed by confirmation via Sanger sequencing. The plasmid maps are given in Figure S2.

#### 2.2.3 Construction of Overexpression Plasmid

The *crtW* gene was synthesized as a gene block, amplified by PCR with primers 25–26, and purified from the gel. The blunt-ended PCR product was first cloned into pJET1.2 (ThermoFisher Scientific) using T4 DNA ligase to yield pNAr11. For the ligation, 50–100 ng vector, insert with a 5:1 vector/insert molar ratio, 1 μL 10× T4 ligase buffer, 1 μL PEG buffer (for blunt-end ligations), 0.5 μL T4 DNA ligase, and dH_2_O up to 10 μL were added. The ligation reactions were incubated on ice for 30 min and then at 18 °C overnight. The constructs pNAr11 and vector pNAr17, which have the pMiS1 vector backbone, were cut by PmeI and SpeI (ThermoFisher Scientific), and the required bands were collected and purified from the gel. A sticky-ended *crtW* fragment obtained from restriction digestion of pNAr11 was inserted into the pMiS1 vector backbone from the restriction digestion of pNAr17 via T4 DNA ligase to yield pNAr18. Constructed plasmids were verified through restriction digestion analysis, followed by confirmation via Sanger sequencing, and their plasmid maps are given in Figure S3.

### 2.3 Transformations and Genetic Engineering of *P. loganensis* sp. nov

Transformations to *P. loganensis* sp. cells were performed via electroporation. Electrocompetent cells were prepared according to the literature.^33^ Electrocompetent cells were prepared by growing cultures to OD_600_ ∼0.4, harvesting on ice, and washing three times with 300 mM sucrose. Cells were resuspended in 100 μL of sucrose, mixed with plasmid DNA, incubated on ice for 10 min, and electroporated at 2.5 kV (12.5 kV/cm) and 25 μF. Cells were immediately recovered in 1 mL of LB at 28 °C for 2 h, then plated on selective LB agar, and incubated at 28 °C for 2 days. Transformants were subcultured and inoculated into the LB with appropriate antibiotics for further use.

For λRed/Cas9-mediated genome editing, a two-step electroporation was performed according to Pfleger and co-workers.^31^ *P. loganensis* sp. nov. cells containing pCas9 were transformed with pJOE and selected on Gen35+Kan30 LB agar plates. Seed cultures were grown and induced with 0.2% l-arabinose at OD_600_ ∼0.4 for 15 min before making cells electrocompetent again for pgRNAtet transformation by the described protocol. The transformants were selected on Gen35+Tet25 plates.

CRISPR-Cas9 plasmids were removed by growth in antibiotic-free LB and repeated plating until no growth occurred on the selective plates. The overexpression plasmid pNAr18 was then introduced into plasmid-free *P. loganensis* sp. nov. ΔcrtX cells, with transformants selected on Kan30 LB agar.

### 2.4 Shake Flask Cultivation for Carotenoid Production

A single colony of the engineered *P. loganensis* sp. nov. strains was separately inoculated into 5 mL of LB medium with appropriate antibiotics and incubated at 28 °C with 220 rpm agitation for 2 days. Shake flask cultures (50, 100, or 200 mL) were started with 0.5% inoculum of the 2 day seed culture, with antibiotics added if required. After 6 h, 0.2% L-rhamnose was added for the induction of astaxanthin production from *P. loganensis* sp. nov. Δ*crtX*/pNAr18. Cultures were incubated at 28 °C with 220 rpm agitation for 5 days before extraction.

### 2.5 PCR Confirmation

Genomic DNAs of wild-type *P. loganensis* sp. nov. and knockout strains were isolated by the genomic DNA extraction kit (Transgen Biotech) and used as templates for the confirmation of knockouts of *crtX*, *crtY*, and *crtZ* genes. PCR was performed on wild-type and knockout genomic DNA with the same primer pair, such as ColonyPCR-CrtX-F and ColonyPCR-CrtX-R (found in Table S1), and a shorter DNA fragment was expected for knockout strains due to gene deletion. The same protocol was applied to the other knockout strains by using the listed primer pairs in Table S1.

### 2.6 Carotenoid Extraction

Five-day cultures were centrifuged at 6000*g* for 10 min to harvest cells. Supernatant was removed, and an equal amount of acetone:methanol:chloroform (4:3:3, v/v/v) mixture was added. Sonication was applied for 1 min per 50 mL of culture to facilitate the extraction of carotenoids, followed by centrifugation at 8000*g* for 15 min to remove cell debris. Supernatants were collected and dried using a rotary evaporator (HeidolpH). The residues were redissolved in acetone:methanol:chloroform (4:3:3) solvent mixture for further analysis.

### 2.7 Thin-Layer Chromatography and High-Performance Liquid Chromatography Analyses

Thin-layer chromatography (TLC) was performed for the separation of carotenoids using silica gel plates coated with the fluorescent indicator F254 (Merck). The developing solvent consisted of acetone:*n*-hexane:dH_2_O (9:9:1, v/v/v). Carotenoid standards, including zeaxanthin, β-carotene, lycopene, and astaxanthin (all purchased from Solarbio), were used as positive controls, while wild-type *P. loganensis* sp. nov. extract served as a negative control.

Extracted carotenoids were further analyzed by high-performance liquid chromatography (HPLC). The analysis was performed on a reverse-phase C18 analytical column (Hypersil ODS, ThermoFisher Scientific; 250 × 4.6 mm, 5 μm particle size). The HPLC instrument (LC-20AD auto sampler, DGU20A_5R_, Shimadzu) was equipped with photodiode array detector (SPD-M20A, Shimadzu) and chromatograms were recorded at 454 nm. The extracted carotenoids were analyzed using an isocratic elution method with acetonitrile:methanol:isopropanol (5:3:2, v/v/v) as the mobile phase, at a flow rate of 1 mL/min and an oven temperature of 40 °C.

### 2.8 Culture Media Optimization Using Response Surface Methodology

A single colony of *P. loganensis* sp. nov. knockout and overexpression strains were separately inoculated into 5 mL of LB medium to obtain the seed cultures. Test tube experiments were then conducted using six different carbon sources (glycerol, rhamnose, sucrose, glucose, fructose, and arabinose) and five nitrogen sources (malt extract, peptone, sodium nitrate, ammonium chloride, and urea). All carbon and nitrogen sources were tested individually. In addition, varying concentrations of carbon and nitrogen sources, different temperatures, and a range of pH values were examined. Each trial was initiated with 1% (v/v) seed culture and performed in independent triplicates. Samples were collected on days 3, 5, and 7 for carotenoid extraction. Extracts were analyzed spectrophotometrically at the specific OD values for each carotenoid, and standard curves were generated using authentic carotenoid standards (Figure S4). Carotenoid concentrations (mg/L) were calculated based on these calibration equations. Area under the curve (AUC) calculations from HPLC analysis were included in the titer calculations to exclude the contribution of impurities and other carotenoids. Graphics were generated and statistical analyses were performed using GraphPad Prism 8.0.2. Based on the *p*-values (*p* < 0.05, *p* < 0.01, *p* < 0.001, and *p* < 0.0001), significance levels were indicated in the graphs using asterisks (*, **, ***, and ****, respectively).

After identifying the most effective carbon and nitrogen sources individually, the response surface methodology (RSM) was applied using Design-Expert 7.0.0 to determine the optimal culture medium for enhancing carotenoid production. A Box–Behnken Design (BBD) was first established to construct a model incorporating multiple experimental factors. The experimental runs generated by the BBD were performed, and the software provided the optimum design parameters. These optimized media compositions were subsequently validated in independent triplicates using 50 mL Falcon tubes containing a 5 mL culture and 250 mL flasks containing a 100 mL culture. In addition, different inoculum volumes (1, 2, 5, and 10%) were tested under the optimized media compositions and conditions. Statistical analysis of the RSM results was performed using Design-Expert version 7.0.0.

## 3 Results

### 3.1 Production of Zeaxanthin via *P. loganensis* sp. nov. Δ*crtX*

In the biosynthetic pathway of wild-type *P. loganensis* sp. nov. (Figure 1), the final product is zeaxanthin diglucoside, which is synthesized from zeaxanthin through the addition of glucose molecules to both β-rings, catalyzed by zeaxanthin glucosyltransferase (CrtX). When the *crtX* gene is knocked out of the genome, the expected final product is zeaxanthin. The knockout of *crtX*, generating the *P. loganensis* sp. nov. Δ*crtX* strain, was confirmed by PCR as described in Section 2.1, and an agarose gel electrophoresis image of the PCR confirmation is seen in Figure S5. Following confirmation, zeaxanthin production was first analyzed using TLC, a cost-effective and rapid chromatographic method for compound separation based on differences in polarity (Figure S6).

The organic carotenoid extract obtained from the cell pellets of *P. loganensis* sp. nov. Δ*crtX* resuspended in an acetone:methanol:chloroform (4:3:3, v/v/v) mixture was further analyzed by HPLC using the described method. The HPLC chromatograms for the extract from the mutant strain and the standard carotenoid are shown in Figure 2. All samples eluted between 3.53 and 3.92 min, forming two distinct peaks. To confirm that these peaks correspond to carotenoids, we examined their UV profiles were examined. Both peaks had nearly identical retention times and UV profiles, with a maximum absorbance at 452 nm, confirming that they represent zeaxanthin isomers. Additionally, the HPLC analysis of *P. loganensis* sp. nov. Δ*crtX* extract revealed an extra peak at 9.64 min, which exhibited a unique UV profile characteristic of lycopene (Figure S7). This finding was consistent with TLC analysis. In the identified carotenoid biosynthetic gene cluster of *P. loganensis* sp. nov., the *crtY* gene is located downstream of the *crtX* gene.^27,28^ Due to the polycistronic nature of bacterial transcription, targeting a single gene can potentially disrupt the transcription and translation of both upstream and downstream genes.^34^ Therefore, the observed accumulation of lycopene in *P. loganensis* sp. nov. Δ*crtX* may be due to impaired *crtY* expression following *crtX* knockout. In the genome, *crtX* and *crtY* are located adjacently, and knocking out *crtX* may have affected *crtY* expression. This effect appears to be partial, as zeaxanthin was still produced; however, *crtY* expression was most likely reduced due to *crtX* deletion since they are located together in one operon.

**Figure 2:**
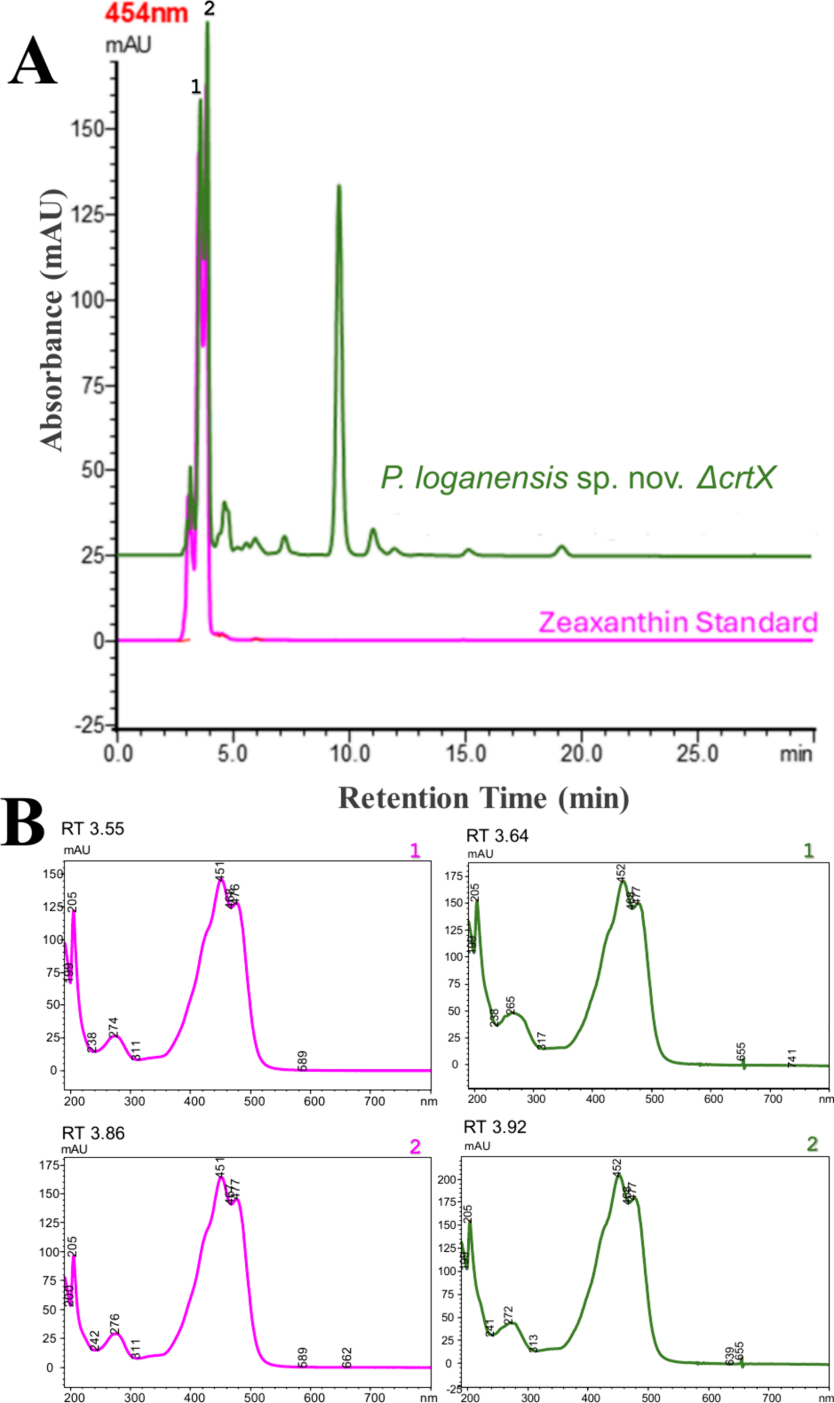
(A) HPLC chromatograms and (B) UV–vis spectral profiles of the peaks for confirmation of *crtX* knockout and zeaxanthin production in *P. loganensis* sp. nov. Δ*crtX*. Chromatogram A (magenta) shows the zeaxanthin standard; chromatogram B (green) shows the carotenoid profile of the *crtX* knockout strain. Peaks labeled 1 and 2 correspond to compounds with similar retention times and UV–Vis spectra in both samples.

To determine the optimal conditions for zeaxanthin production, various parameters—including different carbon sources, nitrogen sources, temperatures, and pH values—were tested individually (Tables S2–S4). Based on titers (mg/L), glycerol (0.5%, seventh day; 4.34 mg/L) and rhamnose (2%, seventh day; 5.76 mg/L) were identified as the most effective carbon sources, while peptone (0.5%, seventh day; 8.96 mg/L) and malt extract (1%, seventh day; 6.90 mg/L) were the most effective nitrogen sources for zeaxanthin production. For subsequent optimization experiments, glycerol and malt extracts were selected. As shown in Figure 6, under standard growth conditions in LB medium, *P. loganensis* sp. nov. *ΔcrtX* produced ∼2.33 mg/L zeaxanthin. Supplementation with 0.5% glycerol increased production to 4.34 mg/L—approximately 2-fold higher than the baseline media—while supplementation with 1% malt extract further enhanced production to 6.90 mg/L, nearly 3-fold higher than under baseline conditions.

To construct a BBD with three independent variables, the glycerol concentration (%), malt extract concentration (%), and incubation time (days) were selected (Table 2). The effects of these parameters on carotenoid production were statistically evaluated, and the optimal media compositions were determined using RSM. The predicted optimum compositions were 0.5% glycerol, 2% malt extract, and 10 days of incubation with a production titer of 15.61 mg/L (Figure S10). Validation experiments were then conducted in Falcon tubes and flasks under the optimized compositions, using different inoculum volumes (1, 2, 5, and 10%; Figure 7). Under these conditions, *P. loganensis* sp. nov. Δ*crtX* produced ∼13.4 mg/L with 1% inoculum in Falcon tubes and ∼11.3 mg/L with 2% inoculum in flasks.

**Table 2:** Box–Behnken Design (BBD) Used for the Optimization of Zeaxanthin, β-Carotene, Lycopene, and Astaxanthin Production in *P. loganensis* sp. nov

glycerol (%)	malt extract (%)	incubation time (days)	zeaxanthin production (mg/L)	beta-carotene production (mg/L)	lycopene production (mg/L)	astaxanthin production (mg/L)
1.25	0.5	5	9.58	20.78	7.75	0.87
1.25	2	5	13.14	27.43	5.45	0.93
0.5	1.25	5	10.75	20.84	8.27	0
2	1.25	5	6.84	27.43	3.81	0.23
1.25	2	10	15.38	17.62	13.31	0
1.25	0.5	10	8.60	16.31	4.49	0.74
2	1.25	10	10.25	20.78	6.34	1.04
0.5	1.25	10	15.84	19.82	8.41	0.05
1.25	1.25	7.5	11.01	25.13	7.99	0
1.25	1.25	7.5	10.99	21.96	7.58	0
1.25	1.25	7.5	10.63	21.37	7.57	0
1.25	1.25	7.5	10.22	20.62	7.70	0
1.25	1.25	7.5	10.54	22.64	8.14	0
2	0.5	7.5	8.43	22.86	7.00	1.51
0.5	2	7.5	12.32	22.37	11.09	0.83
2	2	7.5	13.48	22.51	7.06	1.32
0.5	0.5	7.5	8.00	18.05	7.02	0.69

As shown in Table S2, rhamnose provided an even greater enhancement in zeaxanthin titers in *P. loganensis* sp. nov. Δ*crtX*. However, BBD optimization was not initially performed with rhamnose due to its higher cost compared to glycerol. Despite the economic advantage of glycerol as a carbon source for large-scale applications, BBD was later conducted with three independent variables: rhamnose concentration (%), malt extract concentration (%), and incubation time (days) (Table S14). The effects of these parameters on zeaxanthin production were statistically evaluated, and the optimum media compositions were predicted by RSM as 1.97% rhamnose, 1.98% malt extract, and 9.45 days of incubation, yielding an estimated production titer of 29.99 mg/L (Figure S11). The RSM models (glycerol-based and rhamnose-based) were evaluated for statistical significance. For the model incorporating glycerol, malt extract, and incubation time, the *F*-value was 10.20 with a corresponding *p*-value (Prob > *F*) of 0.001, demonstrating that the model was statistically significant. Similarly, for the model incorporating rhamnose, malt extract, and incubation time, the *F*-value was 44.07 with a *p*-value (Prob > *F*) of <0.0001, also indicating strong statistical significance. Validation experiments under the optimized media conditions were then carried out in shake flasks with varying inoculum volumes (1, 2, 5, and 10%) (Figure S12). The highest titer of 35.0 mg/L zeaxanthin was obtained with a 2% inoculum, corresponding to a 15-fold increase compared to that of the LB base medium.

### 3.2 Production of β-Carotene via *P. loganensis* sp. nov. Δ*crtZ*

β-Carotene is a carotenoid that exhibits an orange color and has pro-vitamin A activity. The hydroxylation of its β-rings by β-carotene hydroxylase (CrtZ) leads to the formation of zeaxanthin. Knocking out the *crtZ* gene in the genome of wild-type *P. loganensis* sp. nov. generated the *P. loganensis* sp. nov. Δ*crtZ* strain, which produces β-carotene as the final product (Figure 1).

Similar to the *crtX* knockout, the knockout of *crtZ* was confirmed by PCR (Figure S5), and the β-carotene production was analyzed using TLC (Figure S6). The organic carotenoid extract from the cell pellets of *P. loganensis* sp. nov. Δ*crtZ* was further analyzed by HPLC. The β-carotene peak eluted between 14.90 and 15.36 min (Figure 3). Both the standard and extract exhibited carotenoid-specific UV profiles with a maximum absorbance at 452 nm. HPLC analysis confirmed both the successful *crtZ* knockout and β-carotene production in the engineered strain. Both β-carotene and lycopene are nonpolar carotenoids, with β-carotene being slightly less polar than lycopene.^35^ As a result, β-carotene typically elutes at a later retention time, depending on the mobile phase. However, due to their minimal polarity difference, lycopene may also elute at a later retention time depending on the polarity of the mobile phase.

**Figure 3:**
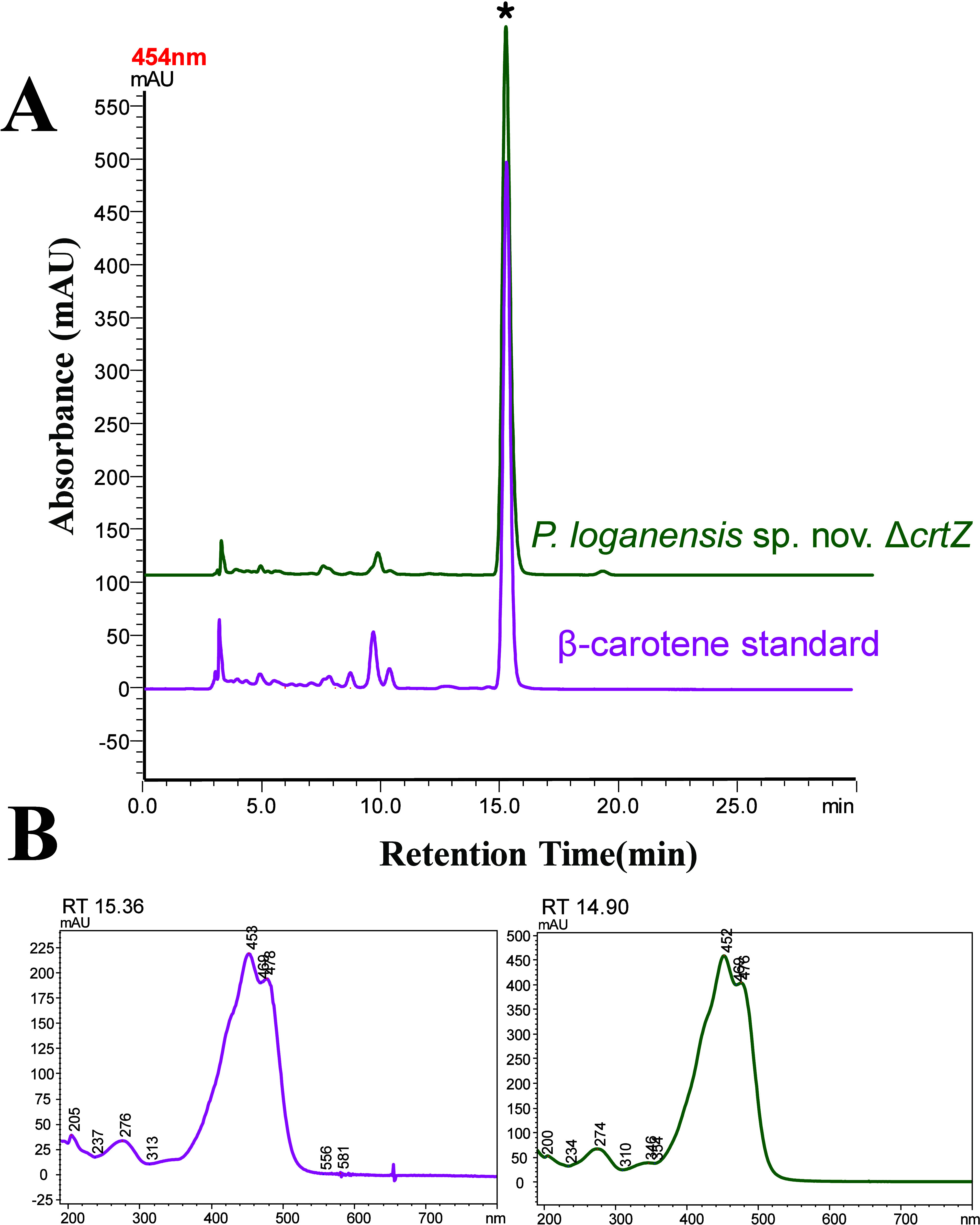
(A) HPLC chromatograms and (B) UV–vis spectral profiles of the peaks for confirmation of *crtZ* knockout and β-carotene production in *P. loganensis* sp. nov. Δ*crtZ*. Chromatogram A (magenta) shows the β-carotene standard; chromatogram B (green) shows the carotenoid profile of the *crtZ* knockout strain. The major peak marked with an asterisk (*) corresponds to β-carotene based on retention time and UV–Vis spectra.

To determine the optimal culture conditions for β-carotene production, various parameters (carbon sources, nitrogen sources, temperatures, and pH values) were tested individually (Tables S5–S7). Among the carbon sources, 0.5% glycerol (seventh day) was the most effective, yielding 19.15 mg/L of β-carotene. Among the nitrogen sources, 1% malt extract (seventh day) was the most effective, with a titer of 17.11 mg/L. As shown in Figure 6, under normal growth conditions in LB media, *P. loganensis* sp. nov. Δ*crtZ* produced ∼4.72 mg/L β-carotene. In contrast, supplementation with either 0.5% glycerol or 1% malt extract increased production nearly 4-fold, reaching 19.15 and 17.11 mg/L, respectively.

To construct the BBD, three independent parameters—glycerol (%), malt extract (%), and incubation time (days)—were selected (Table 2). Experimental runs were performed, and the optimal media compositions were statistically determined using RSM. The optimum compositions for β-carotene production were 2% glycerol, 2% malt extract, and 5 days of incubation with a predicted titer of 27.47 mg/L (Figure S10). The RSM model for β-carotene production was also statistically validated. The model exhibited an *F*-value of 7.44 with a corresponding *p*-value (Prob > *F*) of 0.0038, indicating that the model was statistically significant. Experiments were then conducted in Falcon tubes and shake flasks using different inoculum volumes (1, 2, 5, and 10%) under the optimized media compositions (Figure 7). *P. loganensis* sp. nov. Δ*crtZ* produced approximately 23.53 mg/L β-carotene in Falcon tubes and 21.74 mg/L in flasks, both with a 1% inoculum.

### 3.3 Production of Lycopene via *P. loganensis* sp. nov. Δ*crtY*

Lycopene is the first C40 carotenoid and lacks cyclic groups in its structure (Figure 1). It serves as a precursor for the synthesis of cyclic and bicyclic carotenoids through the catalytic action of lycopene cyclases. Lycopene β-cyclase (CrtY) introduces β-rings at both ends of lycopene, converting it into β-carotene. Since the *crtY* gene was knocked out from the genome of wild-type *P. loganensis* sp. nov., the expected final product is lycopene. The knockout of *crtY* was confirmed by PCR (Figure S5), and the organic carotenoid extract from *P. loganensis* sp. nov. Δ*crtY* was analyzed by TLC, which confirmed the production of lycopene (Figure S6).

The organic carotenoid extract obtained from the cell pellets of *P. loganensis* sp. nov. Δ*crtY* was then analyzed by HPLC, and the resulting chromatogram confirming lycopene production in *P. loganensis* sp. nov. Δ*crtY* is shown in Figure 4. In all samples, the corresponding lycopene peak eluted between 9.34 and 9.54 min. The UV profiles of these peaks are presented in Figure 4. The UV absorption profile exhibited three peaks with maxima at 444, 471, and 503 nm, consistent with lycopene as reported in the literature.^36,37^ These findings confirm the successful knockout of the *crtY* gene and the biosynthesis of lycopene in the engineered strain.

**Figure 4:**
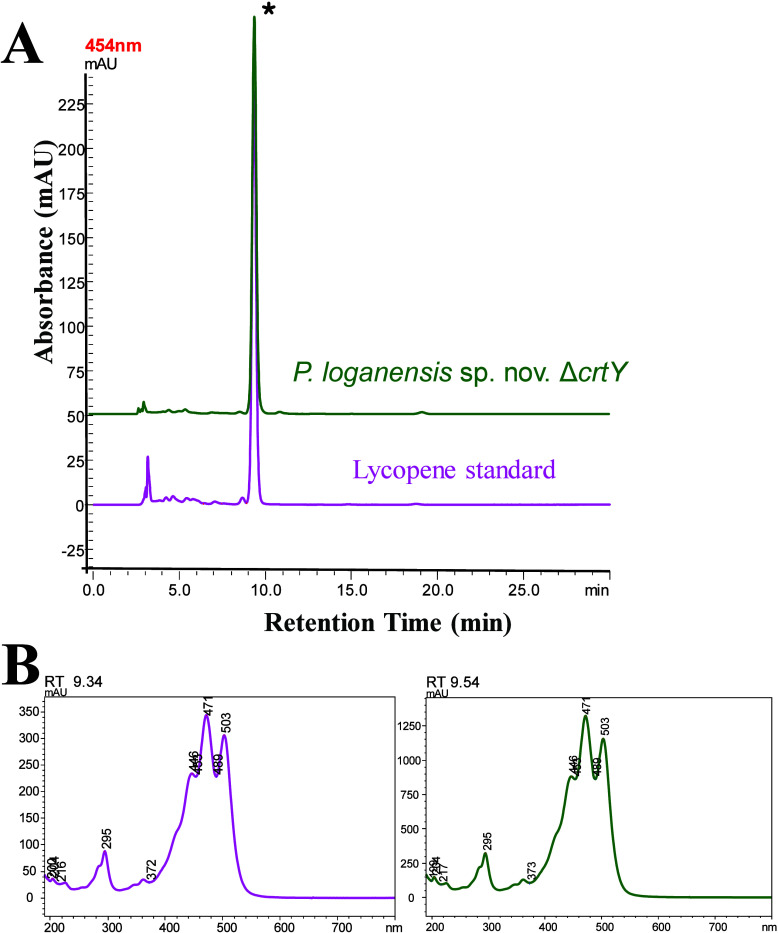
(A) HPLC chromatograms and (B) UV–vis spectral profiles of the peaks for confirmation of *crtY* knockout and lycopene production in *P. loganensis* sp. nov. Δ*crtY*. Chromatogram A (magenta) shows the lycopene standard; chromatogram B (green) shows the carotenoid profile of the *crtY* knockout strain. The major peak marked with an asterisk (*) corresponds to lycopene, based on retention time and UV–Vis absorption.

In the subsequent optimization process for lycopene production, the same parameters were tested individually (Tables S8–S10). Among the carbon sources, glycerol (2%, seventh day) was the most effective, with a titer of 5.47 mg/L lycopene. Similarly, malt extract (1%, seventh day) was identified as the most effective nitrogen source with a titer of 3.95 mg/L lycopene. Under normal growth media, *P. loganensis* sp. nov. Δ*crtY* produced ∼2.02 mg/L lycopene (Figure 6). Supplementation with 2% glycerol increased the titer to 5.47 mg/L, which is approximately twice that under normal conditions, while the addition of 1% malt extract yielded 3.95 mg/L lycopene production, which was also significantly higher than that in the baseline medium.

To further optimize the lycopene production in the engineered strain, a BBD was constructed using three independent parameters: glycerol (%), malt extract (%), and incubation time (days) (Table 2). Experimental runs were conducted, and the results were statistically evaluated using Design-Expert 7.0.0 and used to determine the optimal media compositions through RSM. The optimum media compositions were predicted to be 0.57% glycerol, 1.94% malt extract, and 9.97 days of incubation, with a predicted lycopene titer of 13.5 mg/L (Figure S10). The RSM model for lycopene production was statistically significant, with an *F*-value of 26.75 and a corresponding *p*-value (Prob > *F*) of 0.0001. Validation experiments in Falcon tubes and flasks were carried out with varying inoculum volumes (1, 2, 5, and 10%) under the optimized conditions (Figure 7). *P. loganensis* sp. nov. Δ*crtY* produced ∼9.67 mg/L lycopene in Falcon tubes and ∼8.55 mg/L in flasks, both with a 1% inoculum. Although these were lower than the predicted titer, lycopene production was enhanced by almost 5-fold compared to the base media under normal growth conditions.

### 3.4 Production of Astaxanthin via *P. loganensis* sp. nov. Δ*crtX*/pNAr18

Astaxanthin is a ketocarotenoid synthesized by β-carotene ketolase (CrtW), which catalyzes the conversion of zeaxanthin to astaxanthin (Figure 1). Since the *P. loganensis* sp. nov. Δ*crtX* strain produces zeaxanthin, the introduction of the *crtW* gene via an overexpression plasmid is expected to enable astaxanthin biosynthesis. *P. loganensis* sp. nov. Δ*crtX*/pNAr18 extract was analyzed by TLC (Figure S8) and HPLC along with the astaxanthin standard as a positive control (Figure 5). The astaxanthin standard showed high purity in both TLC and HPLC, with a single peak eluting at 3.65 min. HPLC analysis of *P. loganensis* sp. nov. Δ*crtX*/pNAr18 extract resulted in elution of two peaks: one at 3.70 min, whose profile matched that of the astaxanthin standard with the maximum point at 475 nm. The second peak was eluted with a retention time of 9.29 min, whose UV profile (Figure S9) indicated that it corresponded to lycopene. Lycopene accumulation was first observed in *P. loganensis* sp. nov. Δ*crtX*, likely due to impaired *crtY* expression following *crtX* knockout. Since the astaxanthin-producing strain was constructed from the *crtX* knockout background, lycopene accumulation was also expected. The *crt* gene cluster contains *crtX*, *crtY*, *crtI*, and *crtB* in one operon. The integrity of *crtY* is maintained, as zeaxanthin is still produced; however, its expression level may be reduced due to the *crtX* deletion.

**Figure 5:**
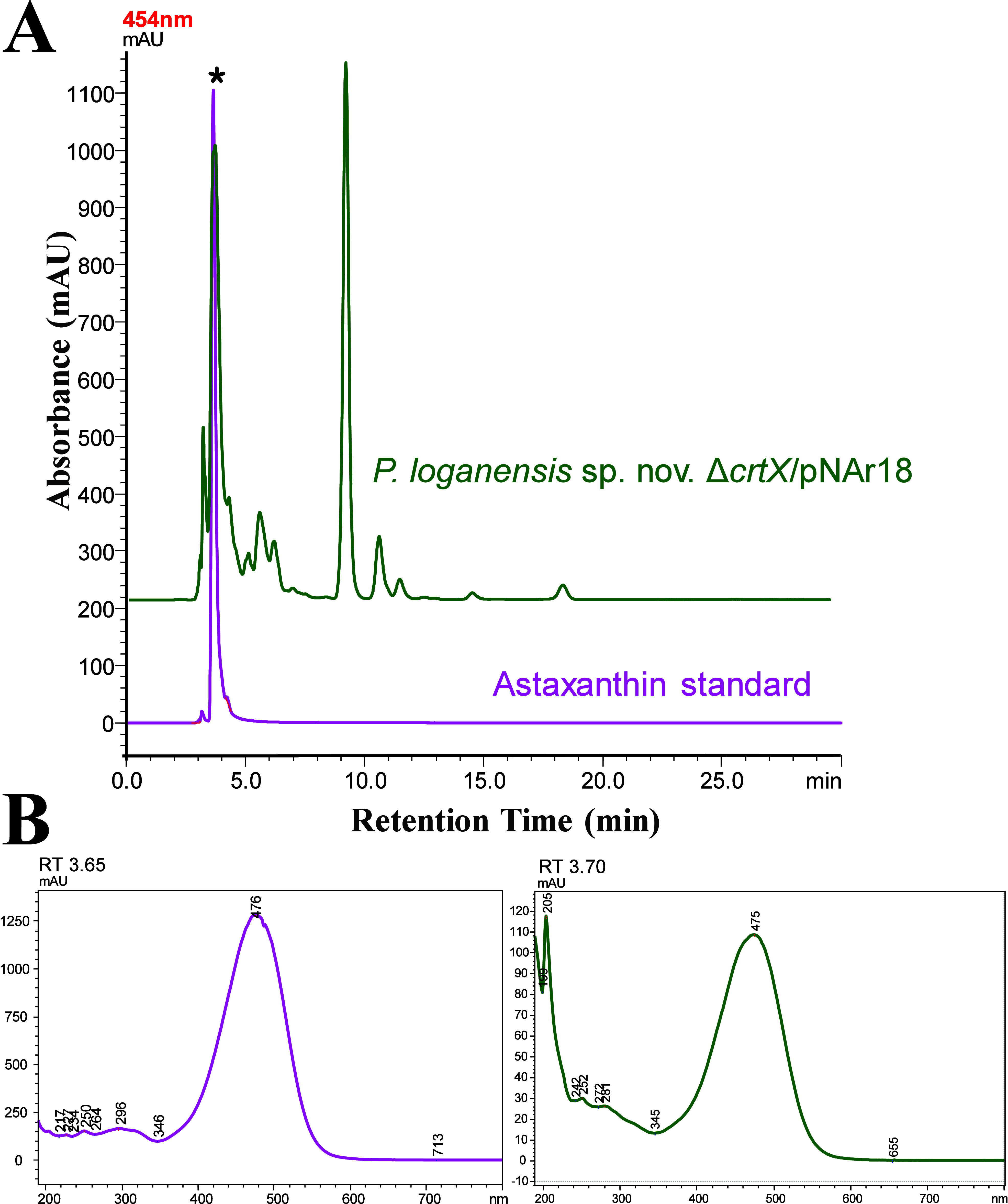
(A) HPLC chromatograms and (B) UV profiles of the peaks for the confirmation of astaxanthin production in *P. loganensis* sp. nov. Δ*crtX*/pNAr18. Chromatogram A (magenta) shows the astaxanthin standard; chromatogram B (green) shows the carotenoid profile of the *crtW* overexpression strain. The major peak marked with an asterisk (*) corresponds to astaxanthin, based on retention time and UV–Vis spectra.

For the optimization of astaxanthin production, various culture parameters—including carbon sources, nitrogen sources, temperatures, and pH—were tested individually (Tables S11–S13). Among the carbon sources, 1% glycerol (seventh day) was the most effective, yielding 0.71 mg/L astaxanthin production, while 2% malt extract (seventh day) was the most effective nitrogen source, with a titer of 0.24 mg/L astaxanthin. Under normal growth conditions with LB base medium, *P. loganensis* sp. nov. Δ*crtX*/pNAr18 produced only ∼0.08 mg/L astaxanthin (Figure 6). Thus, supplementation with glycerol or malt extract significantly increased production compared with that of the baseline medium.

**Figure 6:**
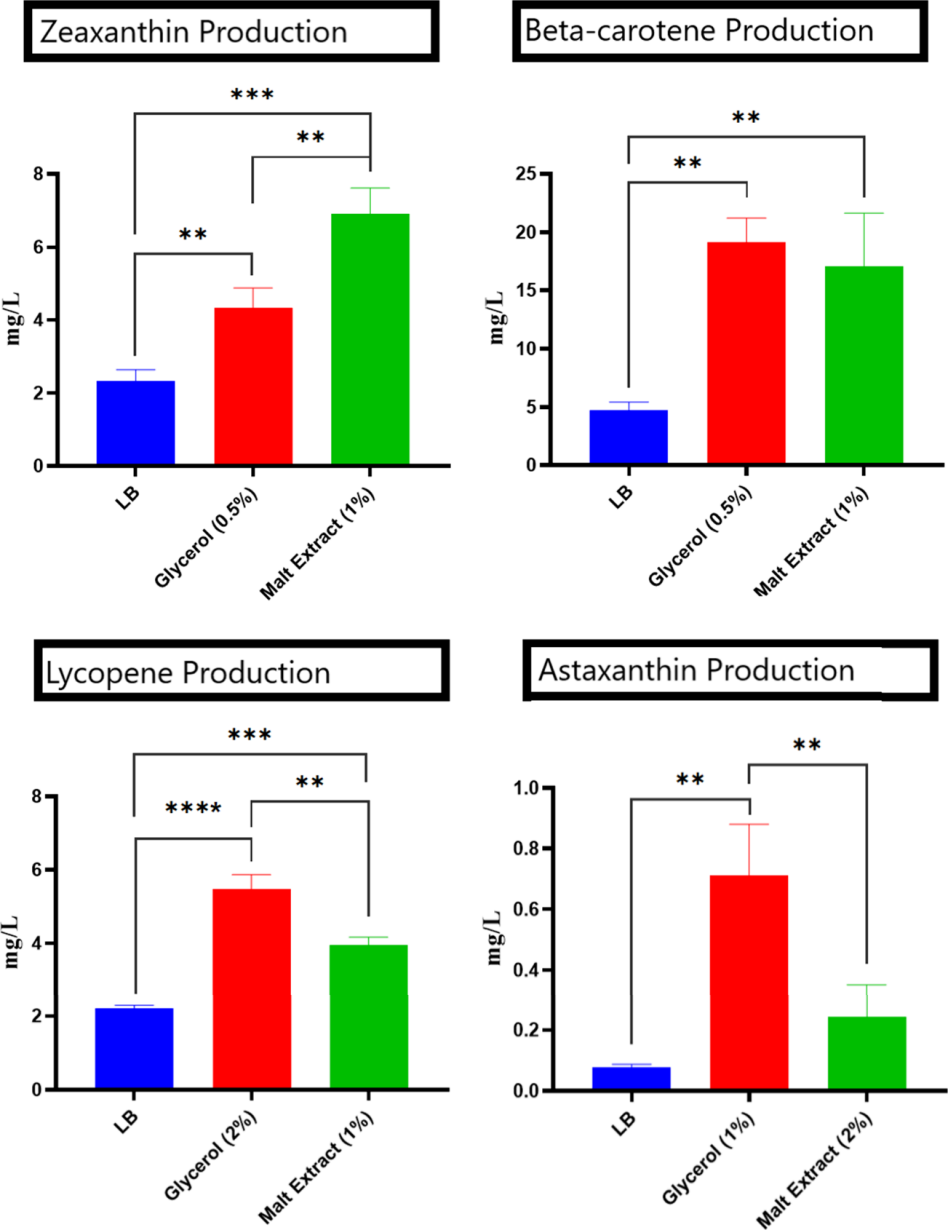
Comparison of carotenoid titer under different media compositions: baseline production in normal growth medium (LB, 7th day, 28 °C), production under the best-performing carbon source, and production under the best-performing nitrogen source.

To further enhance production, a BBD was established using three independent parameters: glycerol (%), malt extract (%), and incubation time (days) (Table 2). Experimental runs were performed, and the results were statistically analyzed to determine the optimal media compositions via RSM. The optimum media compositions were predicted to be 1.96% glycerol, 0.51% malt extract, and 8.82 days of incubation, with a predicted titer of 1.69 mg/L astaxanthin (Figure S10). The RSM model for astaxanthin production was statistically significant, with an *F*-value of 5.57 and a *p*-value (Prob > *F*) of 0.0170. Validation experiments were conducted in Falcon tubes and flasks with varying inoculum volumes (1, 2, 5, and 10%) under optimized culture conditions (Figure 7). *P. loganensis* sp. nov. Δ*crtX*/pNAr18 achieved ∼1.0 mg/L astaxanthin in Falcon tubes and ∼0.8 mg/L in flasks at a 10% inoculum volume. As with lycopene optimization, the measured titers were slightly lower than the predicted value, yet the titer of astaxanthin was enhanced nearly 12-fold compared to that of the base medium under normal growth conditions.

**Figure 7:**
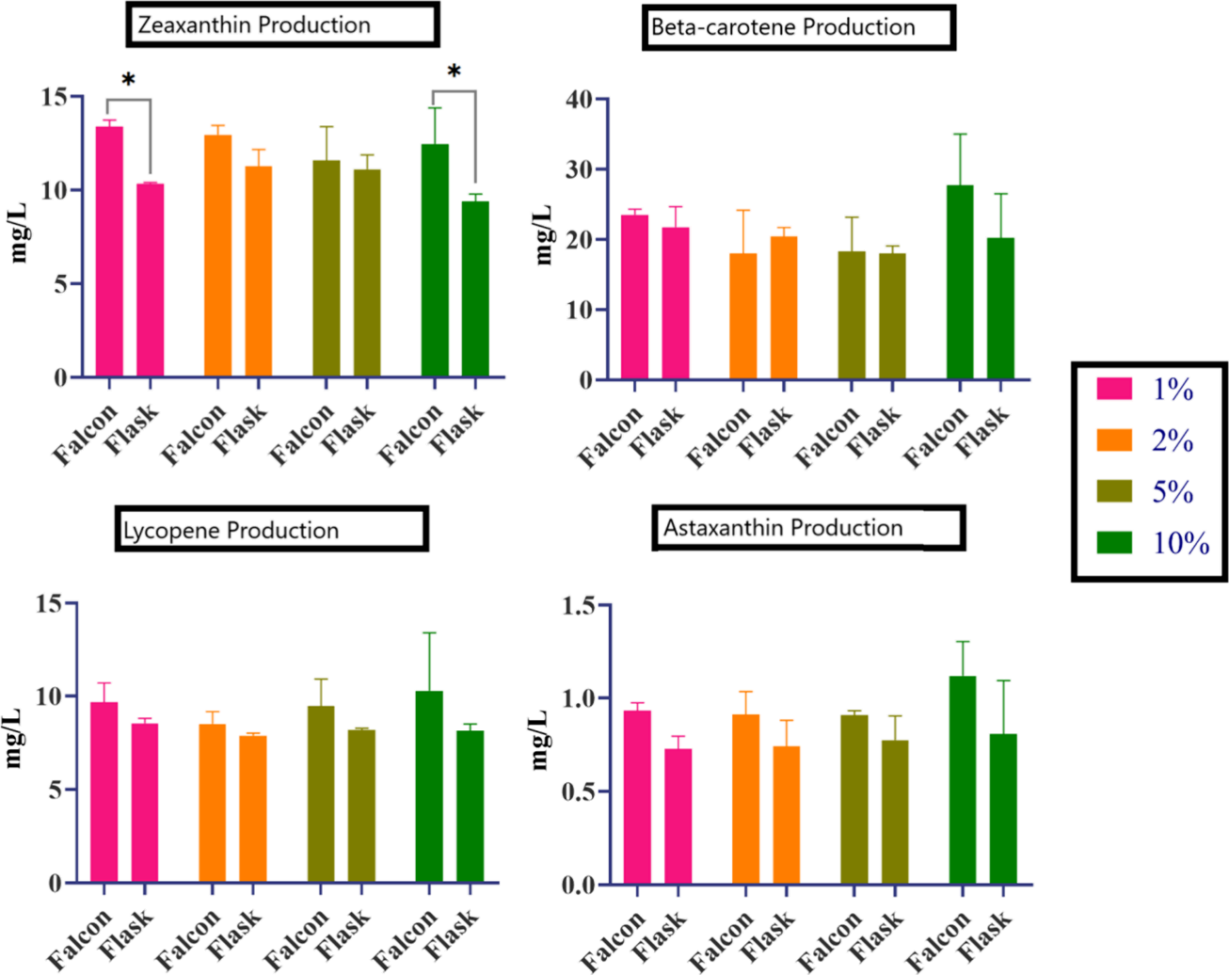
Effect of different inoculum volumes on carotenoid production in Falcon tubes and flasks under optimized medium compositions determined by RSM. Except for the zeaxanthin data, differences in all other carotenoid titers were statistically nonsignificant.

## 4 Discussion

Carotenoids are natural pigments that have various biological activities with diverse applications in the food, feed, cosmetic, and pharmaceutical industries. The demand for carotenoids has increased due to proven benefits to human health.^2,4,38^ Microbial biosynthesis has become a viable and sustainable alternative, both environmentally and economically, to meet the increasing demand.^13^ In this study, we successfully engineered *P. loganensis* sp. nov. to produce four nutritionally valuable carotenoids: lycopene, β-carotene, zeaxanthin, and astaxanthin, using the CRISPR-Cas9 gene editing strategy. Carotenoid biosynthesis starts with isopentenyl diphosphate (IPP) and dimethylallyl diphosphate (DMAPP), and *P. loganensis* sp. nov. natively synthesizes zeaxanthin diglucoside, which is similar, while lycopene, β-carotene, and zeaxanthin are intermediates (Figure 1). By knocking out *crtY*, *crtZ*, and *crtX*, the carotenoid biosynthetic pathway was redirected to accumulate lycopene, β-carotene, and zeaxanthin, respectively. Furthermore, the introduction of the *crtW* gene into the *crtX* knockout strain enabled astaxanthin biosynthesis. TLC and HPLC analyses confirmed the successful production of these target compounds, demonstrating the effectiveness of our genetic modifications. Overall, our findings highlight the potential of *P. loganensis* sp. nov. as a niche microbial chassis for carotenoid biosynthesis and provide a foundation for further metabolic engineering efforts to enhance yields and expand the spectrum of produced carotenoids.

Interestingly, lycopene accumulation was also observed in the zeaxanthin-producing knockout strain. This phenomenon may be explained by a polar effect caused by single gene editing in bacterial operons. It is known that a single-gene deletion may alter the expression of other genes within the same operon in bacterial genomes.^39^ Thus, we hypothesize that the *crtX* knockout might have impaired the expression of *crtY*, leading to reduced expression. However, it could be a partial reduction since the expected carotenoid is still produced. Additionally, RT-qPCR analysis for the expression level of *crtY* might be performed to observe the potential impaired expression. This is a limitation of the current study. To mitigate the polar effect on *crtY*, an additional copy of *crtY* can be introduced to *P. loganensis* sp. nov. Δ*crtX* and lycopene levels can be monitored to determine whether the accumulation persists. Moreover, an independent constitutive promoter and ribosome-binding site (RBS) might be added immediately upstream of *crtY* at the native locus. In addition, scarless or polarity-safe editing of *crtX* could be conducted through base editing or scarless CRISPR to catalytically inactivate *crtX* without disrupting promoter/RBS/translation coupling elements that may affect the expression of *crtY* *P. loganensis* sp. nov.^40,41^

Previously, in the genome of *Pseudomonas putida* KT2440, λ-Red recombineering was shown to enhance the efficiency of homologous recombination for DNA repair.^42^ Based on these findings, Pfleger and co-workers employed λ-Red recombinases, induced by l-arabinose (in pCas9), to improve genome editing efficiency in *P. putida* KT2440.^31^ Furthermore, compared to one-step electroporation, where pJOE and pgRNAtet constructs were transformed at once, two-step electroporation of pJOE and pgRNAtet produced a greater number of transformants with higher editing efficiency.^31^ Considering that this is the first study on genome editing of *P. loganensis* sp. nov., this work demonstrates the ease of genome modification of the endophytic bacteria *P. loganensis* sp. nov. as well as the effectiveness of CRISPR-Cas9 combined with λ-Red recombineering in this species. However, the genome editing efficiency of the CRISPR system still needs to be systematically evaluated.

Various studies have explored the production of carotenoids by bacteria, yeast, and algae. However, only a few studies have utilized endophytic bacterial species for the microbial biosynthesis of carotenoids. In 2023, Hagaggi et al. reported the first β-carotene production as a promising bioactive compound from endophytic bacteria.^43^ Furthermore, another study identified an endophytic marine bacterium that produces astaxanthin, exhibiting cytotoxic activity against the human breast cancer cell line (MCF-7).^44^ Additionally, the wild-type *P. loganensis* sp. nov. was previously identified as a native zeaxanthin diglucoside producer.^28^ Nevertheless, to our knowledge, this is the first study to genetically modify a niche microbial chassis, *P. loganensis* sp. nov., to produce four different carotenoids, revealing the untapped potential of endophytic bacteria as microbial cell factories for the production of valuable food coloring agents.

In this study, carotenoid production was substantially increased through individual carbon and nitrogen tests combined with RSM optimization. Particularly, glycerol as the carbon source and malt extract as the nitrogen source consistently enhanced the titer of the carotenoids in the engineered strains. The glycerol can be assimilated into the central metabolic pathways after its phosphorylation. Glycerol was found to be efficiently channeled to biomass accumulation through the glyoxylate shunt in *P. putida* KT2440.^45,46^ A similar regulon is present in *P. loganensis* sp. nov. genome, indicating its direct utilization of glycerol as a carbon source. Malt extract contains various carbon sources, including maltose and glucose, as well as protein.^47^ In particular, glucose can be directly utilized to support biomass accumulation, which, in turn, may enhance carotenoid production.

RSM optimization strategies have been widely used in previous research. For instance, zeaxanthin production was optimized in freshwater isolates by adjusting pH, temperature, inoculum size, agitation speed, carbon source, and harvest time with *Arthrobacter gandavensis* MTCC 25325 after OFAT and RSM optimization.^48^ In addition, Plackett-Burman design (PBD) and RSM optimization of medium composition (1.4 g/L glucose, 26.5 g/L peptone, pH 8.5, 30 °C) enhanced β-carotene production in *Exiguobacterium acetylicum* S01 3.47-fold, reaching ∼40.32 mg/L.^49^ In *Arthrobacter agilis* A17 (KP318146), BBD and RSM optimization with molasses, yeast extract, and KH_2_PO_4_ resulted in ∼100 mg/L β-carotene, a 2.5-fold increase over the baseline medium. This approach demonstrated the efficiency of inexpensive carbon sources combined with statistical design.^50^ In another study, RSM-mediated medium optimization enhanced the titer of astaxanthin 5-fold in batch cultures (39.6 mg/L) and 2.5-fold in fed-batch fermentation (176 mg/L) in *Corynebacterium glutamicum*.^51^ Similarly, *Phaffia rhodozyma* D3 achieved a ∼3-fold higher astaxanthin production (4.70 mg/g) with an optimized medium containing 32 g/L glucose, 12 g/L corn steep liquor, and pH 6.7.^52^ In the present study, zeaxanthin, lycopene, and β-carotene titers were improved ∼5-fold through RSM optimization, while astaxanthin production was enhanced ∼12-fold compared to that in the LB media without optimization. The zeaxanthin titer was further enhanced with the utilization of rhamnose as a carbon source by ∼15-fold (Table 3). Collectively, these results, together with prior reports, highlight the significance of RSM in optimizing culture conditions and media compositions and its importance for enhanced carotenoid production. However, these results are limited to experiments in shake flasks and Falcon tubes. Thus, demonstrations under bioreactor conditions are required to evaluate the industrial relevance and scalability.

**Table 3:** Summary of the Carotenoid Production Titer of Mutant and Overexpression Strains in This Study

strains	titer in LB media (mg/L)	titer in optimized media (mg/L)	carbon sources (%)	nitrogen sources (%)	incubation time (day)	factor of increase in titer
*P. loganensis* sp. nov. Δ*crtX* (zeaxanthin)	2.33	13.40	0.5 glycerol	2 malt extract	10 days	5.75
*P. loganensis* sp. nov. Δ*crtX* (zeaxanthin)	2.33	35.00	1.97 rhamnose	1.98 malt extract	9.45 days	15.02
*P. loganensis* sp. nov. Δ*crtZ* (β-carotene)	4.72	23.53	2 glycerol	2 malt extract	5 days	4.99
*P. loganensis* sp. nov. Δ*crtY* (lycopene)	2.02	9.67	0.57 glycerol	1.94 malt extract	9.97 days	4.79
*P. loganensis* sp. nov. Δ*crtX/*pNAr18 (astaxanthin)	0.08	1.0	1.96 glycerol	0.51 malt extract	8.82 days	12.50

With the advances in synthetic biology, particularly certain host strains such as *E. coli*, *S. cerevisiae*, and *Yarrowia lipolytica* have been extensively engineered to generate high producer strains for the economically viable microbial production of nutritionally valuable carotenoids. In this sense, *E. coli* was engineered for astaxanthin biosynthesis by combining *crtZ* genes with different substrate preferences, resulting in the titer of 1.82 g/L astaxanthin after 70 h of fed-batch fermentation.^53^ Another study employed an integrated approach including systems metabolic engineering, cell morphology engineering, inner- and outer-membrane vesicle (IMV and OMV) formation, and fermentation optimization to produce a spectrum of pigments in *E. coli*. This strategy led to the titers of 322 mg/L astaxanthin, 343 mg/L β-carotene, 218 mg/L zeaxanthin, 1.42 g/L proviolacein, 0.844 g/L prodeoxyviolacein, 6.19 g/L violacein, and 11.26 g/L deoxyviolacein.^54^ In *S. cerevisiae*, one study enhanced β-carotene production by coexpressing lipases and carotenogenic genes with a 12-fold increase and reached 477.9 mg/L β-carotene in the YPD medium supplemented with 1% (v/v) olive oil as the carbon source.^55^ Another study in *S. cerevisiae* implemented multiple metabolic engineering strategies that boosted β-carotene production by ∼5-fold with a titer of ∼167 mg/L β-carotene in shake flasks.^56^ Similarly, metabolic engineering and pathway optimization in *Y. lipolytica* enabled the production of 2.7 g/L β-carotene under fed-batch fermentation conditions.^57^ In another study, the substrate inhibition of lycopene cyclase was mitigated through protein engineering for carotenoid biosynthesis in *Y. lipolytica*. With the additional metabolic engineering strategies, the titers of β-carotene and lycopene reached up to 39.5 and 8.02 g/L, respectively, in bioreactor fermentations.^58^ *Y. lipolytica* was also engineered for zeaxanthin production. The systematic metabolic engineering strategies were employed to enhance the titer of zeaxanthin to 729 mg/L in shake flasks and 2.55 g/L in a batch bioreactor.^59^

Overall, our engineered strains achieved titers comparable to those reported for some microbial systems. Nevertheless, the carotenoid yields remain substantially lower than those in optimized industrial hosts. Thus, this study should be regarded as a proof of concept that establishes *P. loganensis* sp. nov. as a genetically tractable and niche microbial chassis rather than as directly competitive with current microbial platforms. Carotenoid overproduction can impose a substantial metabolic burden on the host and consume considerable cellular resources, which can limit the overall productivity. Therefore, an additional pathway refinement is necessary. Carotenoids are synthesized from IPP and DMAPP, which are derived from either the mevalonate (MVA) or the 2-*C*-methyl-d-erythritol 4-phosphate (MEP) pathways. Enhancing the supply of metabolic precursors should be prioritized to increase the flux toward carotenoid production. Introducing and optimizing the corresponding genes from the MVA or MEP pathways may further enhance the titers of the carotenoids across all engineered strains. Alongside pathway engineering, adaptive laboratory evolution (ALE) could be employed to strengthen genetic stability and boost the carotenoid yield. The combination of rational metabolic balancing and ALE may improve strain robustness and make the engineered strains more suitable for industrial applications. Building on these strategies, the incorporation of synthetic biology tools could further enhance carotenoid production. CRISPRi offers a means to fine-tune gene expression and minimize pathway competition without introducing permanent genetic changes. Additionally, the modular pathway design enables separate construction and optimization of carotenoid biosynthetic genes and precursor supply pathways, allowing each module to be individually enhanced for maximal efficiency. Furthermore, bioreactor trials will be essential to assessing oxygen transfer, shear stress tolerance, and nutrient utilization under controlled conditions for industrial scalability. Together, these approaches can provide a comprehensive framework for strain optimization and future large-scale deployment.

Recent studies have highlighted the necessity of integrating life cycle assessment (LCA) and techno-economic analysis (TEA) in the assessment of microbial carotenoid production systems. For instance, comprehensive analyses involving LCA and TEA were conducted to evaluate carotenoid production from *P. rhodozyma* yeast, considering factors like solvent selection and extraction methods.^60^ Similarly, Ferreira et al. performed LCA and TEA to assess the impacts of cultivation reactors and extraction methods for carotenoid production from wastewater-grown microalgae biomass.^61^ Incorporating such analyses in future studies of *P. loganensis* sp. nov. would provide a more comprehensive understanding of its sustainability and economic feasibility.

## Supplementary Material



## Notes

Plasmid maps of the constructed suicide plasmids with homologue arms for the *crtX*, *crtY*, and *crtZ* knockouts, pNar7, pNar10, and pNar14, respectively (Figure S1); plasmid maps of the constructed guide RNA plasmids containing N20 PAM sequences for the *crtX*, *crtY*, and *crtZ* knockouts, pNar8, pNar9, and pNar15, respectively (Figure S2); plasmid maps of the constructed cloning and expression plasmids for *crtW* and *CaZEP* genes, pNar11­(cloning plasmid with pJET1.2 vector), pNar17 (*CaZEP* in pMiS1 expression plasmid) and pNar18 (*crtW* in pMiS1 expression plasmid) (Figure S3); standard curves for zeaxanthin, β-carotene, lycopene, and astaxanthin (Figure S4); agarose gel electrophoresis results for PCR confirmation of knockout strains (Figure S5); TLC analysis of extracts from knockouts with positive (carotenoid standard) and negative (wild type *P. loganensis* sp. nov. extract) controls (Figure S6); UV profile of the peak at 9.64 min from HPLC chromatogram of *P. loganensis* sp. nov. Δ*crtX* extract (Figure S7); TLC analysis of extracts from overexpression strain *P. loganensis* sp. nov. Δ*crtX*/pNAr18 with positive (astaxanthin standard) and negative (*P. loganensis* sp. nov. Δ*crtX* extract) controls (Figure S8); UV profile of the peak at 9.29 min from HPLC chromatogram of *P. loganensis* sp. nov. Δ*crtX*/pNAr18 extract (Figure S9); RSM analysis showing the effect of glycerol and malt extract on the production of different carotenoids (zeaxanthin, β-carotene, lycopene, and astaxanthin) (Figure S10); RSM analysis showing the effect of rhamnose and malt extract on the production of zeaxanthin (Figure S11); effect of different inoculum volumes in flasks under optimized medium media (rhamnose as the carbon source) determined by RSM for zeaxanthin production (Figure S12); list of all the primers used in this study (Table S1); individual test tube trials with different carbon sources for zeaxanthin production (Table S2); individual test tube trials with different nitrogen sources for zeaxanthin production (Table S3); individual test tube trials with different temperature and pH settings for zeaxanthin production (Table S4); individual test tube trials with different carbon sources for β-carotene production (Table S5); individual test tube trials with different nitrogen sources for β-carotene production (Table S6); individual test tube trials with different temperature and pH settings for β-carotene production (Table S7); individual test tube trials with different carbon sources for lycopene production (Table S8); individual test tube trials with different nitrogen sources for lycopene production (Table S9); individual test tube trials with different temperature and pH settings for lycopene production (Table S10); individual test tube trials with different carbon sources for astaxanthin production (Table S11); individual test tube trials with different nitrogen sources for astaxanthin production (Table S12); individual test tube trials with different temperature and pH settings for astaxanthin production (Table S13); Box–Behnken design (BBD) used for the optimization of zeaxanthin using rhamnose as carbon source (Table S14) (PDF)
