# Supplementary material for: CRISPR/Cas9-Mediated Metabolic Engineering of Endophytic Pseudomonas loganensis sp. nov. for the Production of Nutritionally Valuable Carotenoids
Source: ACS Omega. 2026 Jan 2;11(1):535–51. doi: 10.1021/acsomega.5c05877 (PMC12809803; doi:10.1021/acsomega.5c05877)
Supplement: Supplementary file 1 [file ao5c05877_si_001.pdf]

Supporting Information for

CRISPR/Cas9-Mediated Metabolic Engineering of Endophytic *Pseudomonas loganensis* sp. nov. for the Production of Nutritionally Valuable Carotenoids

Nuriye Arslansoy[#]

Affiliation: Department of Bioengineering, Graduate School of Science and Engineering, Abdullah Gül University, Kayseri, Turkiye

Melisa Zulal Karaman[#]

Affiliation: Department of Bioengineering, Graduate School of Science and Engineering, Abdullah Gül University, Kayseri, Turkiye

Ozkan Fidan*

Affiliation: Department of Bioengineering, Faculty of Natural and Life Sciences, Abdullah Gül University, Kayseri, Turkiye

Telephone: +90 352 2248800

E-mail: ozkan.fidan@agu.edu.tr

[#] These authors contribute equally.

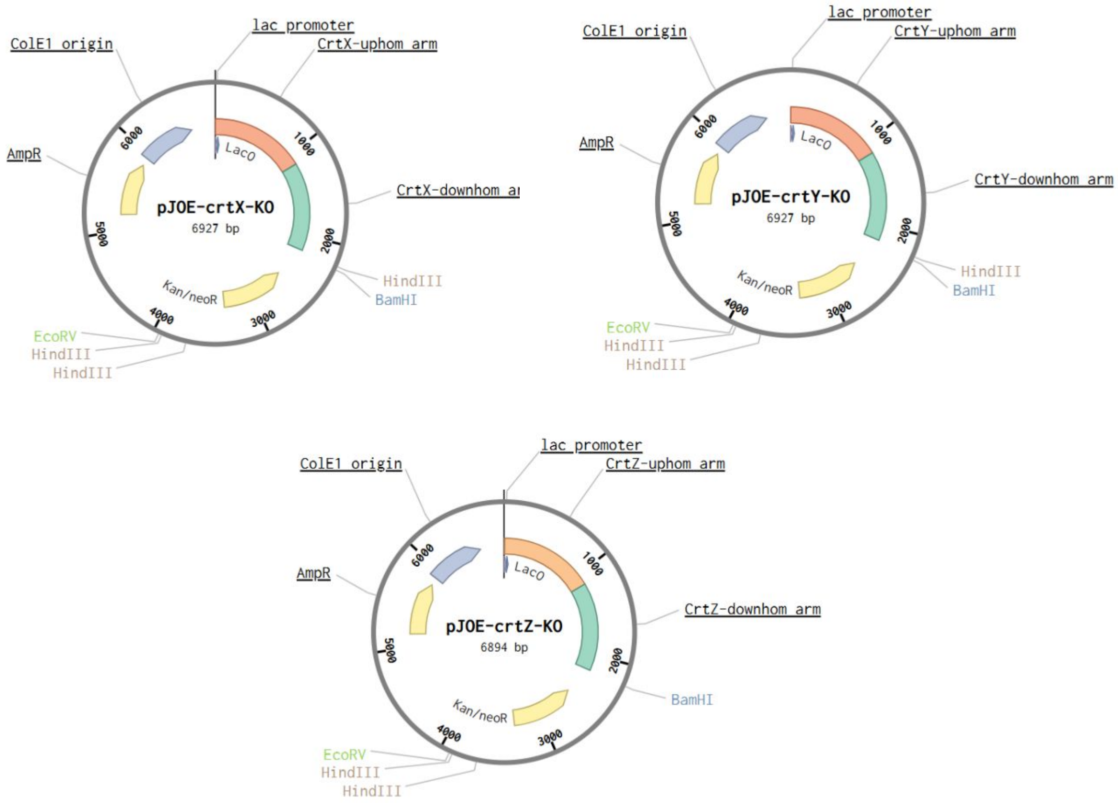

Figure S1. Plasmid maps of the constructed suicide plasmids with homolog arms for the *crtX*, *crtY*, and *crtZ* knockouts, pNar7, pNar10 and pNar14, respectively.

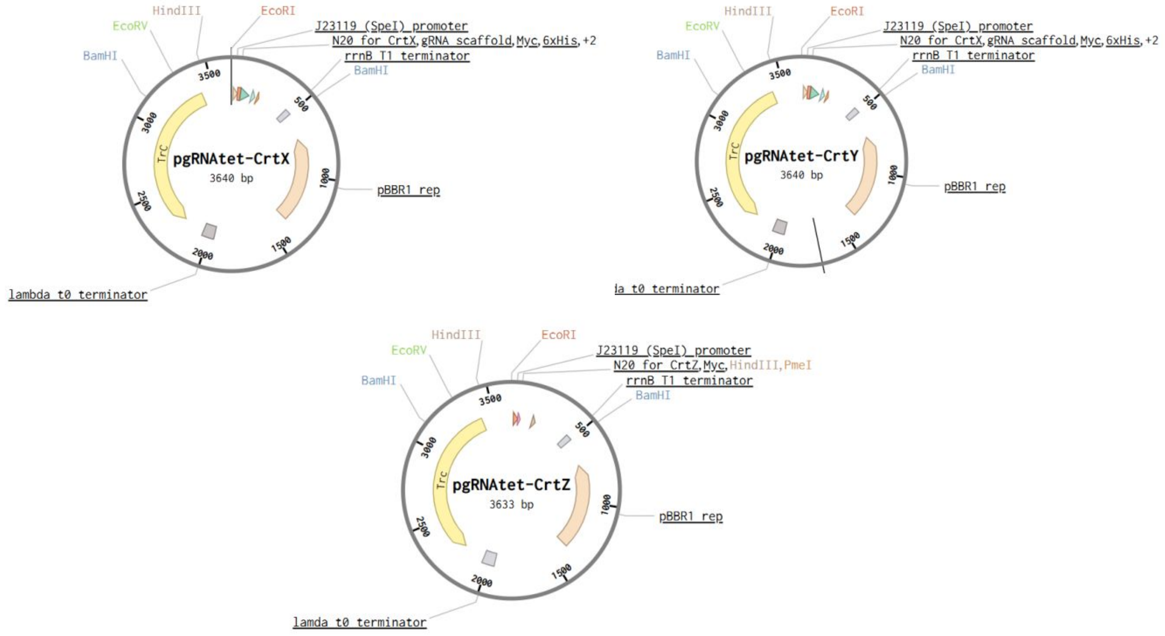

Figure S2. Plasmid maps of the constructed guide RNA plasmids containing N20 PAM sequences for the *crtX*, *crtY*, and *crtZ* knockouts, pNar8, pNar9 and pNar15, respectively.

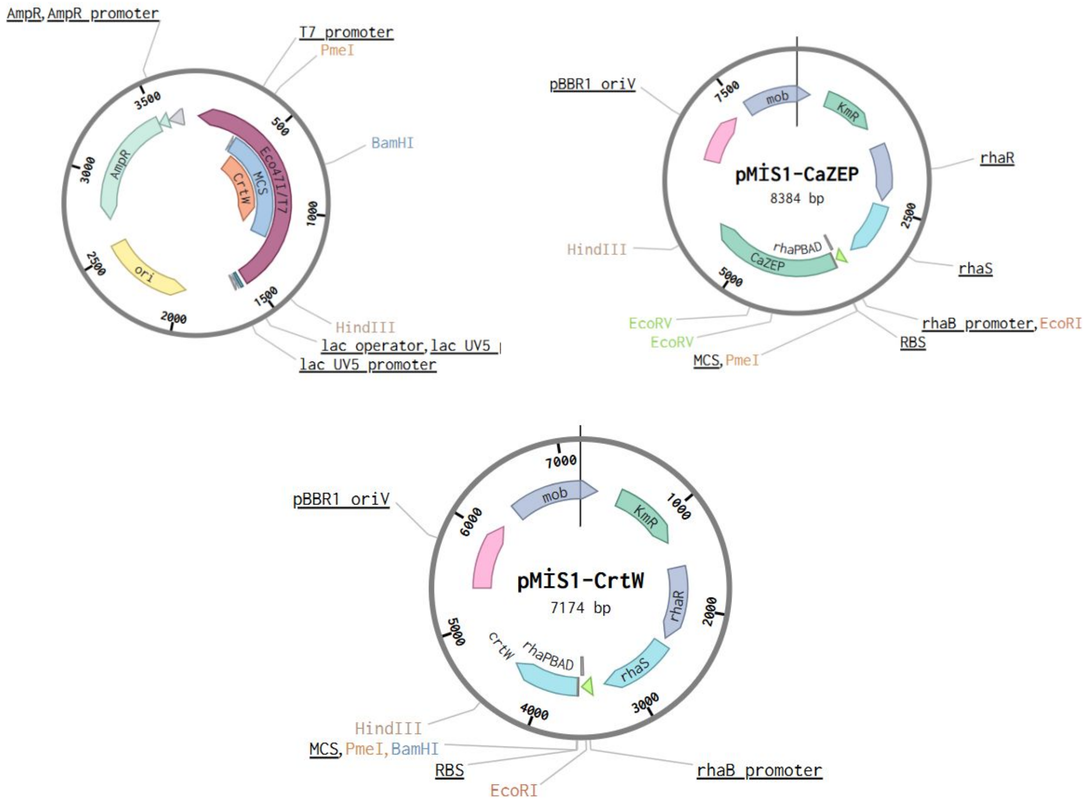

Figure S3. Plasmid maps of the constructed cloning and expression plasmids for *crtW* and *CaZEP* genes, pNar11(cloning plasmid with pJET1.2 vector), pNar17 (*CaZEP* in pMiS1 expression plasmid) and pNar18 (*crtW* in pMiS1 expression plasmid).

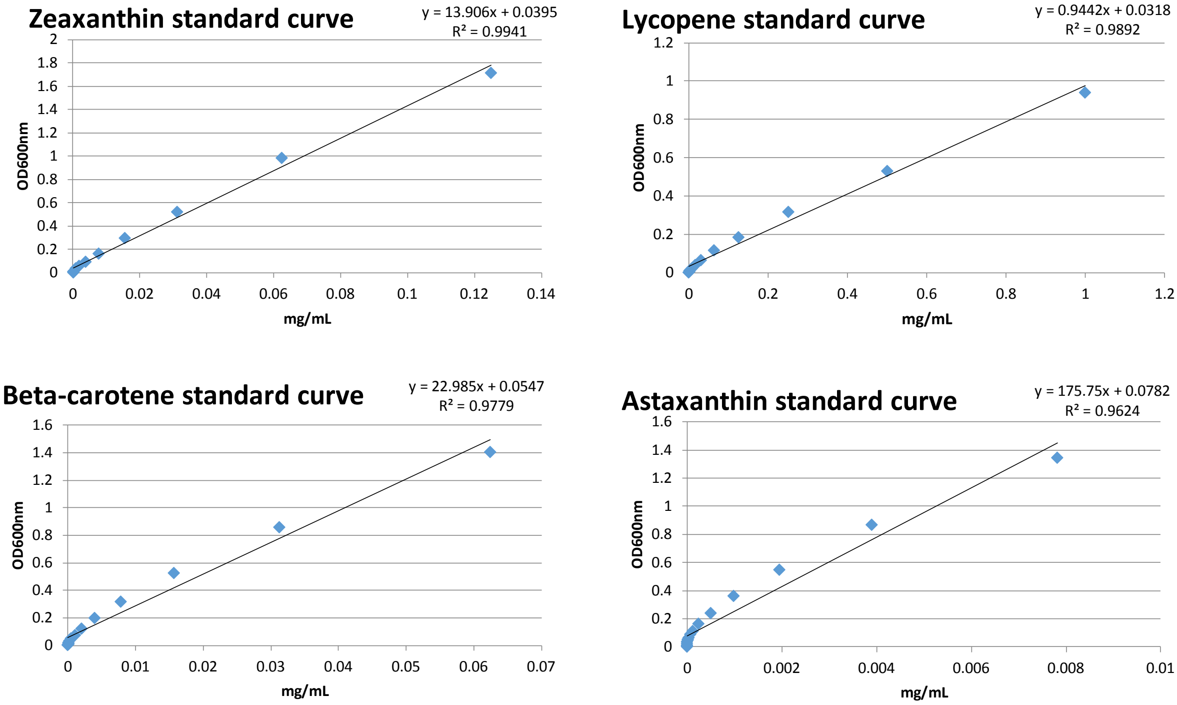

Figure S4. Standard curves for zeaxanthin, β-carotene, lycopene, and astaxanthin.

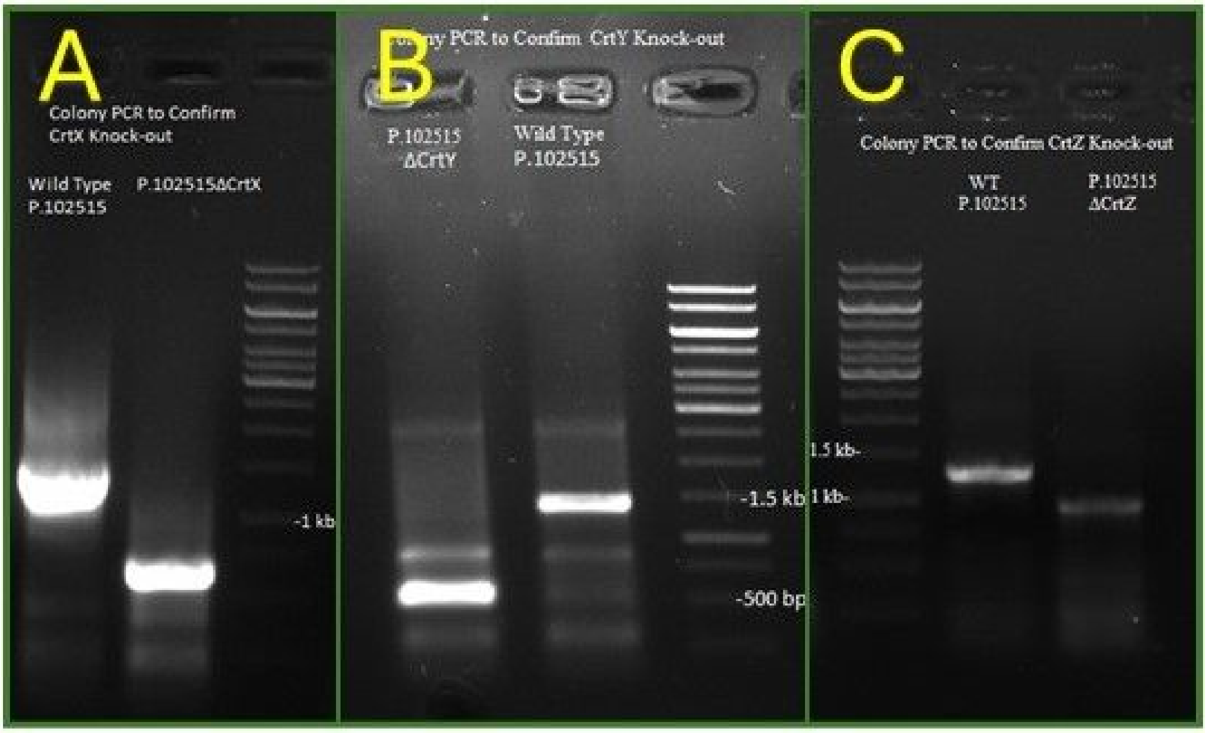

Figure S5. Agarose gel electrophoresis result for PCR confirmation of knockout strains. A) Confirmation of Δ*crtX*, B) Confirmation of Δ*crtY*, C) Confirmation of Δ*crtZ*.

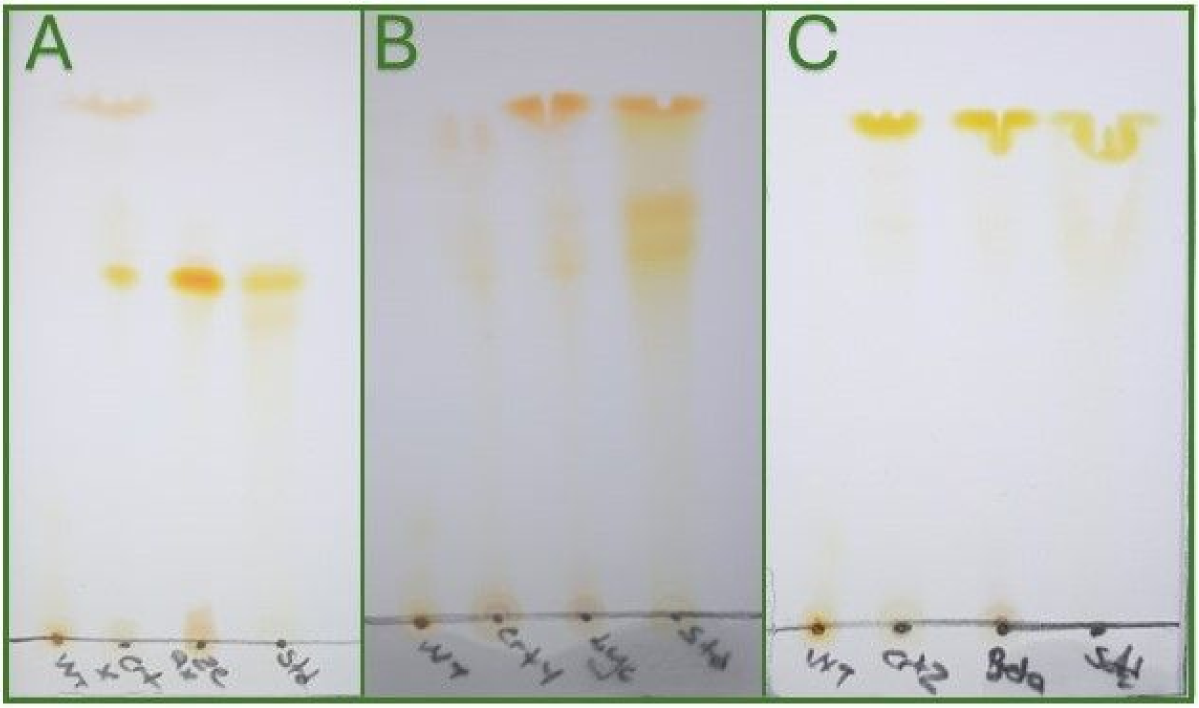

Figure S6. TLC analysis of extracts from knockouts with positive (carotenoid standard) and negative (wild type *Pseudomonas loganensis* sp. nov. extract) controls. A) TLC for extract of *P. loganensis* sp. nov. Δ*crtX*, B) TLC for extract of *P. loganensis* sp. nov. Δ*crtY*, C) TLC for extract of *P. loganensis* sp. nov. Δ*crtZ.*

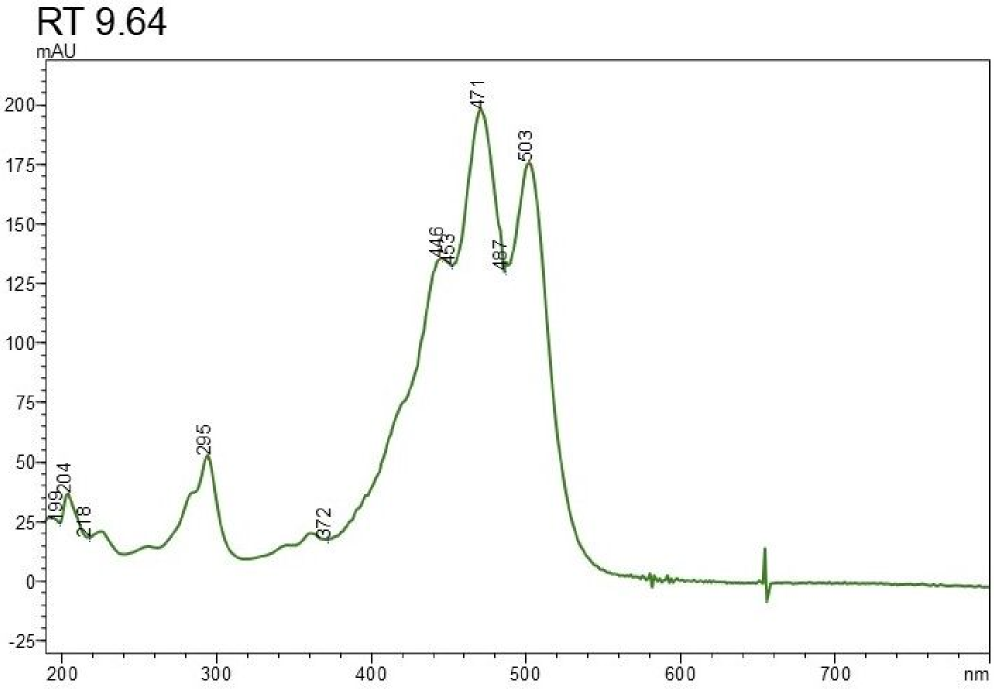

Figure S7. UV profile of the peak at 9.64 min from HPLC chromatogram of *P. loganensis* sp. nov. Δ*crtX* extract.

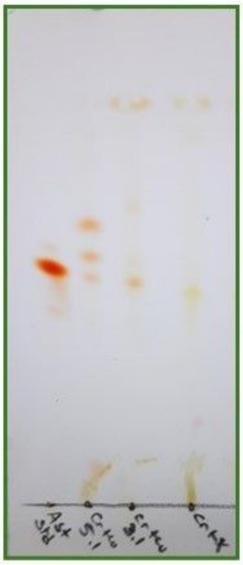

Figure S8. TLC analysis of extracts from overexpression strain *P. loganensis* sp. nov. Δ*crtX* /pNAr18 with positive (astaxanthin standard) and negative (*P. loganensis* sp. nov. Δ*crtX* extract) controls.

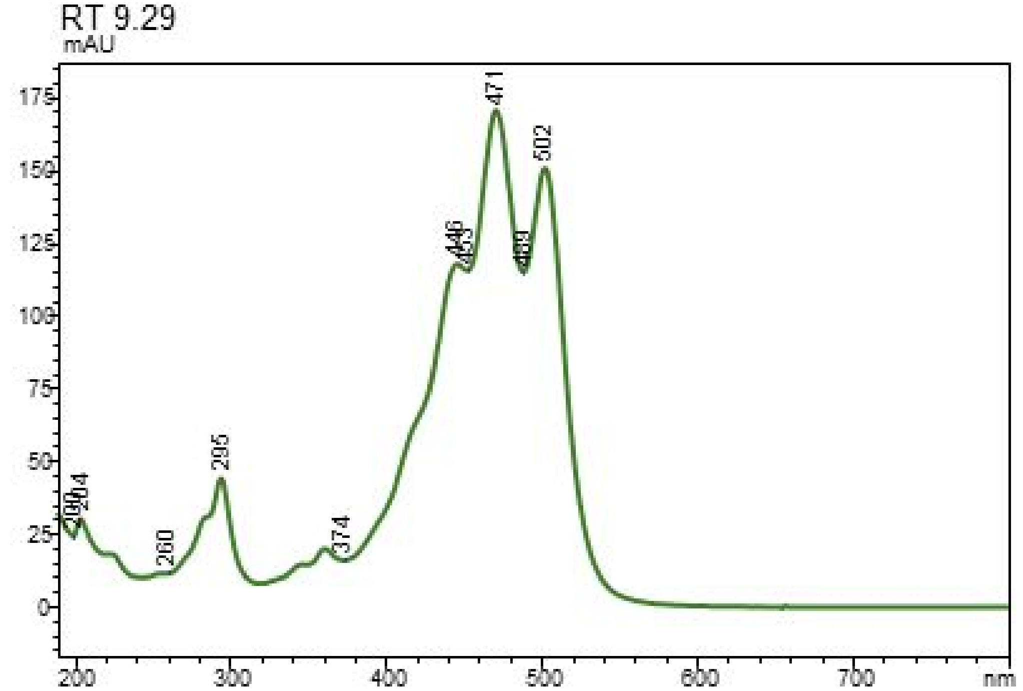

Figure S9. UV profile of the peak at 9.29 min from HPLC chromatogram of *P. loganensis* sp. nov. Δ*crtX*/pNAr18 extract.

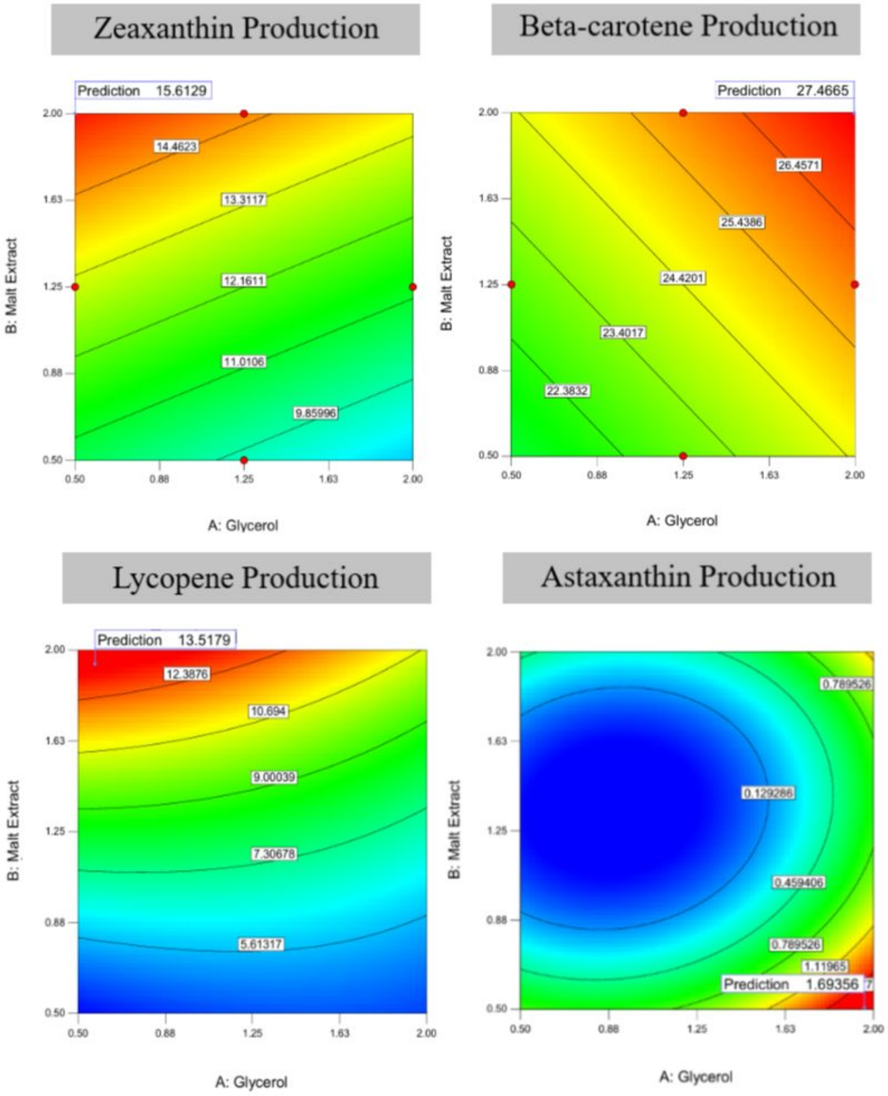

Figure S10. RSM analysis showing the effect of glycerol and malt extract on the production of different carotenoids (zeaxanthin, β-carotene, lycopene, and astaxanthin).

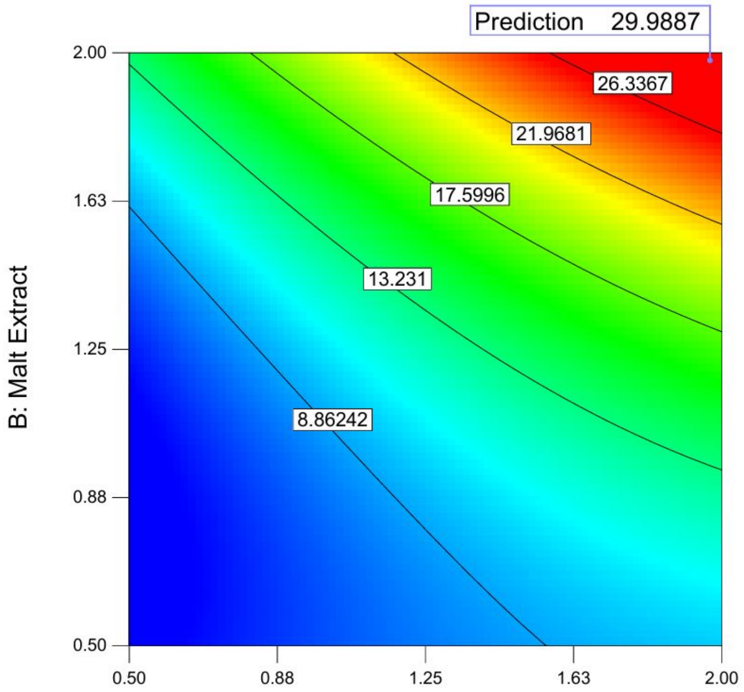

Figure S11. RSM analysis showing the effect of rhamnose and malt extract on the production of zeaxanthin.

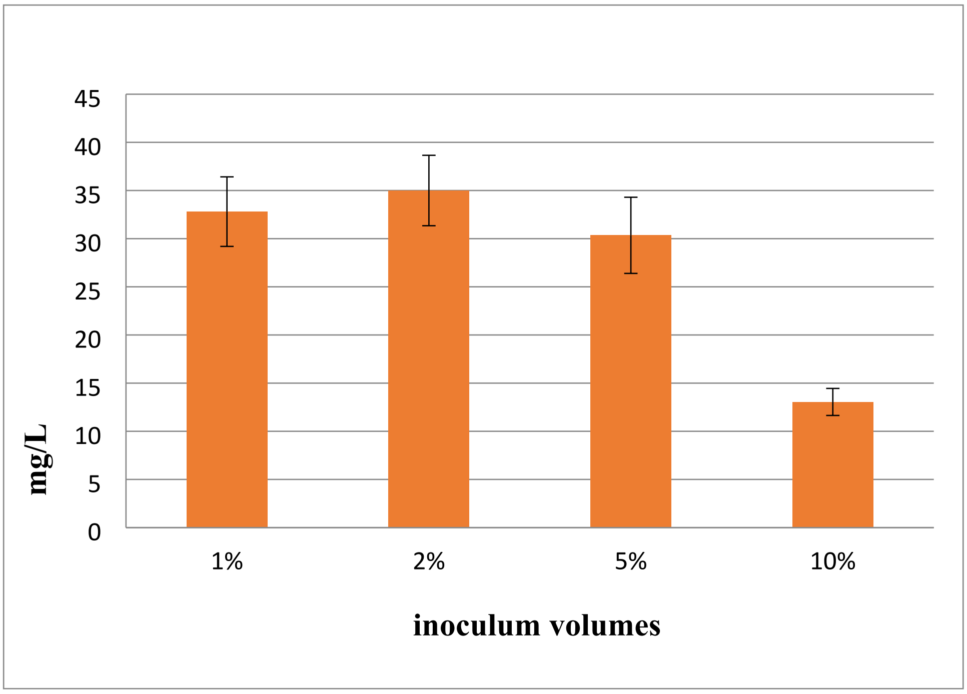

Figure S12. Effect of different inoculum volumes in flasks under optimized medium media (rhamnose as the carbon source) determined by RSM for zeaxanthin production.

Table S1. List of all the primers used in this study.

Primer No.	Oligo Name	Oligonucleotide Sequence 5'-3'
1	CrtX-Uphom-F-PmlI	AACACGTGACGCCCTGTTCTTCGCGCAGCA
2	CrtX-Uphom-R	CGACATGGCGCGCAACTGCCCATGGTGTTCGACCA
3	CrtX-Downhom-F	GAACACCATGGGCAGTTGCGCGCCATGTCGGCGATCA
4	CrtX-Downhom-R-BamHI	AAGGATCCATCGTGCATCTCAATCCC
5	CrtY-Uphom-F-PmlI	TGCACGTGCAGCACCAGCACGTGGTGGCGCGATTGCCTTCG
6	CrtY-Uphom-R	AGGTGCGCTTATCCTCTCCGGCAAACCGC
7	CrtY-Downhom-F	CGGAGAGGATAAGCGCACCTCGTAGCCG
8	CrtY-Downhom-R-BamHI	GGCCGCTTTGGTCCCGGATCCAGGTGTTCGACCTGATGATGCG
9	CrtZ-Uphom-F-PmlI	TGCACGTGCAGCACCAGCACGTGCTCAATGGCAAGGAAACCGG
10	CrtZ-Uphom-R	GGAAACCGAAGCATGATGTACTTGTGCGACC
11	CrtZ-Downhom-F	TACATCATGCTTCGGTTTCCTGCTGGCG
12	CrtZ-Downhom-R-BamHI	GGCCGCTTTGGTCCCGGATCCGCGATAAACGGGTCGGATTA
13	pgRNAtet-CrtX-F	AGGTATAATACTAGTGCTGGCGGTACGCGCCAGCAGGTTTTAGAGCTAGA
14	pgRNAtet-F	GCTTGGATTCTCACCAATAAAAAAC
15	pgRNAtet-R	ACTAGTATTATACCTAGGACTGAGC
16	pgRNAtet-CrtXYZ-R	GGTGAGAATCCAAGCGCCTCCGCCCTGCGGCCT
17	pgRNAtet-CrtY-F	AGGTATAATACTAGTCTGTTCTTCGCGCAGCACCTGTTTTAGAGCTAGA
18	pgRNAtet-CrtZ-F	AGGTATAATACTAGTCCCTGGGCAATGCCGGCTATGTTTTAGAGCTAGA
19	ColonyPCR-CrtX-F	AGTCCAGCTCCGGACGGGTTTG
20	ColonyPCR-CrtX-R	TGCGGTGCCCATAGTGGCCGACA
21	ColonyPCR-CrtY-F	AGGGTGGTGGCGATGCCGGC

22	ColonyPCR-CrtY-R	GCCGAACGCTTCGTGCCCAT
23	ColonyPCR-CrtZ-F	CGAATGGGAAGGCGAATTCC
24	ColonyPCR-CrtZ-R	TCGATCCCTTCGACGAGCAG
25	CrtW-OE-PmeI-F	AAGTTTAAACATGGTCCAGTGCCAGCCGTC
26	CrtW-OE-SpeI-R	AAACTAGTTTACAGCGAGATTTTGTGTGCTT

Table S2. Individual test tube trials with different carbon sources for zeaxanthin production.

Carbon Sources							
**Days**		**Glycerol**	**Glucose**	**Fructose**	**Sucrose**	**Rhamnose**	**Arabinose**
**3rd day**	**0,5%**	-0,09±0,40	-0,10±0,25	0,51±0,07	2,75±0,57	1,11±0,31	0,23±0,21
	**1%**	0,17±0,47	0,02±0,21	-0,17±0,10	0,54±0,73	1,52±0,32	-0,52±0,12
	**2%**	-0,16±0,05	-0,59±0,14	0,23±0,33	1,28±0,26	0,80±0,40	-1,00±0,09
**5th day**	**0,5%**	1,94±0,33	1,26±0,61	2,62±0,18	1,25±0,61	3,00±0,54	1,87±0,33
	**1%**	1,42±0,41	0,22±0,55	1,29±0,19	2,55±0,22	4,41±0,66	-0,15±0,83
	**2%**	0,82±0,21	-0,72±0,23	0,76±0,32	2,05±0,24	4,81±1,48	-0,94±0,07
**7th day**	**0,5%**	4,34±0,44	1,56±0,23	2,82±0,04	1,45±0,93	2,38±0,41	3,12±0,71
	**1%**	2,82±0,19	2,33±0,30	2,75±0,21	2,15±0,79	4,69±0,39	2,35±0,11
	**2%**	2,42±0,28	-0,48±0,09	1,19±0,26	2,61±0,45	5,76±2,22	-0,68±0,21

Table S3. Individual test tube trials with different nitrogen sources for zeaxanthin production.

Nitrogen Sources						
**Days**		**Sodium Nitrate**	**Ammonium Chloride**	**Peptone**	**Malt Extract**	**Urea**
**3rd day**	**0,5%**	1,03±0,15	-0,43±0,31	2,80±0,62	0,72±0,73	-0,16±0,25
	**1%**	0,31±0,52	-0,79±0,14	1,07±1,26	1,07±0,93	-0,43±0,11
	**2%**	-0,44±0,21	-1,15±0,09	0,97±0,63	0,87±0,49	-0,64±0,05
**5th day**	**0,5%**	0,13±0,19	-0,43±0,43	3,24±0,65	2,26±0,78	-1,15±0,27
	**1%**	-0,38±0,20	-1,00±0,14	1,45±1,34	2,19±1,30	-1,49±0,03
	**2%**	-1,46±0,05	-1,36±0,09	1,92±0,40	2,16±0,429	-1,51±0,09
**7th day**	**0,5%**	1,85±0,25	0,36±0,18	8,96±0,99	6,46±0,68	0,27±0,33
	**1%**	0,73±0,76	-0,29±0,14	6,04±1,79	6,90±0,58	-0,16±0,07
	**2%**	-0,41±0,13	-0,76±0,12	6,04±0,37	5,86±1,20	-0,45±0,11

Table S4. Individual test tube trials with different temperature and pH settings for zeaxanthin production.

Different temperature and pH settings						
**Days**	**18 °C**	**28 °C**	**37 °C**	**pH 5**	**pH 7**	**pH 9**
**3rd day**	-0,13±0,07	0,85±0,39	-0,92±0,12	-0,72±0,07	-0,09±0,27	0,13±0,23
**5th day**	0,43±0,07	1,53±0,39	-0,66±0,16	-0,19±0,07	0,29±0,17	0,37±0,22
**7th day**	0,76±0,31	2,33±0,26	-0,27±0,21	0,34±0,14	1,07±0,09	0,73±0,42

Table S5. Individual test tube trials with different carbon sources for β-carotene production.

Carbon Sources							
**Days**		**Glycerol**	**Glucose**	**Fructose**	**Sucrose**	**Rhamnose**	**Arabinose**
**3rd day**	**0,5%**	6,82±1,47	0,40±0,20	5,44±0,52	2,66±0,03	3,44±0,91	1,62±0,39
	**1%**	5,,56±0,50	1,15±0,05	6,31±0,19	2,9±0,25	3,23±0,10	1,64±0,05
	**2%**	4,84±0,66	0,80±0,18	5,35±0,16	1,99±0,10	4,39±1,99	0,25±0,03
**5th day**	**0,5%**	11,03±2,21	0,50±0,29	14,27±1,37	4,11±0,81	4,23±1,43	4,54±0,28
	**1%**	6,31±0,79	1,44±0,12	8,31±0,25	4,97±0,59	4,82±0,15	1,80±0,19
	**2%**	5,96±0,62	1,21±0,05	6,84±0,25	4,25±0,03	5,86±2,38	0,86±0,08
**7th day**	**0,5%**	19,15±1,69	2,01±0,90	15,86±1,20	6,64±0,58	6,11±0,30	8,13±0,67
	**1%**	12,03±1,88	2,48±0,25	16,23±0,32	6,76±0,29	6,05±0,10	1,56±0,21
	**2%**	10,33±1,44	2,87±0,53	8,11±0,33	5,80±1,40	5,70±2,45	0,93±0,10

Table S6. Individual test tube trials with different nitrogen sources for β-carotene production.

Nitrogen Sources						
**Days**		**Sodium Nitrate**	**Ammonium Chloride**	**Peptone**	**Malt Extract**	**Urea**
**3rd day**	**0,5%**	3,11±0,18	5,25±1,78	12,96±1,03	13,58±0,87	5,09±0,72
	**1%**	4,31±1,85	5,92±0,05	12,90±0,44	12,94±2,33	2,76±0,26
	**2%**	0,21±0,10	2,23±0,59	15,27±0,20	9,84±0,53	1,66±0,25
**5th day**	**0,5%**	3,78±1,06	5,62±1,15	13,21±0,80	14,88±2,32	5,90±1,48
	**1%**	4,25±1,51	6,15±0,28	12,13±0,54	13,07±2,60	2,64±0,20
	**2%**	0,13±0,12	3,33±0,81	14,64±0,54	11,11±3,28	2,07±0,54
**7th day**	**0,5%**	8,72±0,64	2,48±0,26	15,74±0,41	15,37±2,71	6,31±1,62
	**1%**	4,64±1,70	1,56±0,21	13,49±2,27	17,11±3,70	6,74±1,27
	**2%**	0,31±0,08	0,86±0,18	14,66±1,79	13,68±2,29	3,86±0,38

Table S7. Individual test tube trials with different temperature and pH settings for β-carotene production.

Different temperature and pH settings						
**Days**	**18 °C**	**28 °C**	**37 °C**	**pH 5**	**pH 7**	**pH 9**
**3rd day**	0,72±0,26	3,74±0,68	0,74±0,59	-0,28±0,03	6,07±1,01	-0,28±0,03
**5th day**	2,66±0,45	4,44±0,86	0,95±0,45	-0,26±0,05	5,72±0,62	-0,32±0,00
**7th day**	3,68±0,48	4,72±0,59	1,60±0,45	-0,21±0,08	5,68±0,91	-0,01±0,05

Table S8. Individual test tube trials with different carbon sources for lycopene production.

Carbon Sources							
**Days**		**Glycerol**	**Glucose**	**Fructose**	**Sucrose**	**Rhamnose**	**Arabinose**
**3rd day**	**0,5%**	1,24±0	1,36±0,10	1,37±0,21	2,30±0,53	1,30±0,35	0,66±0,03
	**1%**	1,40±0,11	1,45±0,07	2,08±0,15	2,89±0,27	1,43±0,06	0,60±0,05
	**2%**	1,27±0,06	1,51±0,04	1,27±0,16	2,06±0,77	1,18±0,17	0,70±0,28
**5th day**	**0,5%**	1,52±0,10	2,14±0,72	3,82±0,57	2,77±0,66	1,55±0,33	1,42±0,16
	**1%**	1,85±0,28	1,31±0,21	2,53±0,44	3,47±0,22	1,67±0,29	0,73±0,03
	**2%**	1,60±0,08	1,61±0,07	1,18±0,58	2,26±1,04	1,70±0,05	0,60±0,04
**7th day**	**0,5%**	4,92±0,75	3,51±0,40	4,03±0,61	2,77±0,62	1,24±0,20	2,52±0,43
	**1%**	3,97±0,81	1,34±0,19	5,04±0,80	3,57±0,35	1,98±0,22	0,73±0,03
	**2%**	5,47±0,32	1,43±0,01	1,73±0,29	2,77±0,66	2,19±0,09	0,51±0,03

Table S9. Individual test tube trials with different nitrogen sources for lycopene production.

Nitrogen Sources						
**Days**		**Sodium Nitrate**	**Ammonium Chloride**	**Peptone**	**Malt Extract**	**Urea**
**3rd day**	**0,5%**	1,61±0,10	0,92±0,02	1,15±0,02	1,23±0,04	1,54±0,13
	**1%**	1,13±0,06	0,70±0,08	1,51±0,13	1,27±0,06	1,67±0,11
	**2%**	0,43±0,02	0,41±0,02	1,24±0,18	1,38±0,12	0,92±0,26
**5th day**	**0,5%**	2,39±0,18	1,38±0,11	2,56±0,19	3,24±0,46	1,34±0,33
	**1%**	1,48±0,08	1,08±0,08	2,51±0,02	2,05±0,52	1,64±0,02
	**2%**	0,59±0,01	0,55±0,02	2,44±0,03	2,29±0,20	0,86±0,21
**7th day**	**0,5%**	2,70±0,22	2,18±0,11	2,76±0,11	3,60±0,57	2,89±0,41
	**1%**	1,40±0,08	1,59±0,14	2,79±0,09	3,95±0,17	1,85±0,12
	**2%**	0,35±0,02	0,50±0,04	2,69±0,13	3,65±0,10	1,04±0,17

Table S10. Individual test tube trials with different temperature and pH settings for lycopene production.

Different temperature and pH settings						
**Days**	**18 °C**	**28 °C**	**37 °C**	**pH 5**	**pH 7**	**pH 9**
**3rd day**	0,64±0,08	1,47±0,32	0,40±0,03	0,33±0,01	1,88±0,16	0,33±0,01
**5th day**	0,85±0,14	1,75±0,14	0,51±0,10	0,32±0,02	2,01±0,14	0,32±0,00
**7th day**	1,01±0,06	2,02±0,07	0,64±0,02	0,32±0,02	2,11±0,15	0,35±0,01

Table S11. Individual test tube trials with different carbon sources for astaxanthin production.

Carbon Sources							
**Days**		**Glycerol**	**Glucose**	**Fructose**	**Sucrose**	**Rhamnose**	**Arabinose**
**3rd day**	**0,5%**	0,12±0,11	-0,06±0,03	0,26±0,03	-0,03±0,01	0,01±0,03	-0,01±0,05
	**1%**	0,12±0,02	-0,08±0,02	0,11±0,02	-1,14±0,01	0,03±0,10	-0,15±0,15
	**2%**	-0,02±0,02	-0,10±0,02	0,08±0,01	-0,07±0,01	-0,07±0,07	-0,16±0,01
**5th day**	**0,5%**	0,26±0,12	0,13±0,06	0,29±0,02	0,20±0,14	0,06±0,05	0,19±0,09
	**1%**	0,07±0,04	-0,1±0,05	0,32±0,07	-0,14±0,01	0,20±0,12	-0,20±0,02
	**2%**	-0,01±0,01	-0,13±0,01	0,15±0,03	-0,10±0,01	0,31±0,15	-0,22±0,01
**7th day**	**0,5%**	0,55±0,10	0,35±0,01	0,50±0,04	0,,42±0,01	0,18±0,05	0,31±0,07
	**1%**	0,71±0,14	-0,07±0,01	0,58±0,04	-0,11±0,02	0,34±0,13	-0,12±0,02
	**2%**	0,54±0,02	-0,04±0,04	0,32±0,10	-0,07±0,01	0,36±0,03	-0,14±0,01

Table S12. Individual test tube trials with different nitrogen sources for astaxanthin production.

Nitrogen Sources						
**Days**		**Sodium Nitrate**	**Ammonium Chloride**	**Peptone**	**Malt Extract**	**Urea**
**3rd day**	**0,5%**	-0,05±0,06	-0,08±0,04	-0,06±0,01	-0,05±0,02	-0,06±0,02
	**1%**	-0,06±0,03	-0,16±0,01	-0,06±0,05	-0,05±0,03	-0,11±0,02
	**2%**	-0,18±0,01	-0,20±0,02	-0,06±0,02	-0,01±0,02	-0,18±0,01
**5th day**	**0,5%**	0,04±0,05	0,02±0,06	0,01±0,01	0,08±0,02	0,01±0,02
	**1%**	0,02±0,04	-0,11±0,01	0,06±0,01	0,09±0,04	-0,02±0,02
	**2%**	-0,11±0,01	-0,18±0,02	0,07±0,05	0,23±0,08	-0,14±0,02
**7th day**	**0,5%**	0,19±0,04	0,09±0,07	0,13±0,03	0,21±0,01	0,15±0,04
	**1%**	0,06±0,01	-0,10±0,01	0,16±0,04	0,17±0,04	0,06±0,02
	**2%**	-0,15±0,01	-0,14±0,02	0,17±0,01	0,24±0,09	0,05±0,05

Table S13. Individual test tube trials with different temperature and pH settings for astaxanthin production.

Different temperature and pH settings						
**Days**	**18 °C**	**28 °C**	**37 °C**	**pH 5**	**pH 7**	**pH 9**
**3rd day**	-0,05±0,10	-0,08±0,02	-0,22±0,01	-0,24±0,00	-0,07±0,00	-0,24±0,00
**5th day**	-0,15±0,01	0,01±0,01	-0,05±0,03	-0,2±0,00	0,03±0,01	-0,23±0,01
**7th day**	-0,08±0,01	0,08±0,03	-0,04±0,03	-0,22±0,00	0,08±0,01	-0,21±0,00

Table S14. Box–Behnken design (BBD) used for the optimization of zeaxanthin using rhamnose as carbon source.

**Rhamnose(%)**	**Malt Extract (%)**	**Incubation Time (Days)**	**Zeaxanthin Production (mg/L)**
1,25	0,5	5	7,16208
1,25	2	5	20,4241
0,5	1,25	5	7,55337
2	1,25	5	10,8933
1,25	2	10	24,5327
1,25	0,5	10	7,44157
2	1,25	10	18,0205
0,5	1,25	10	7,24593
1,25	1,25	7,5	9,25829
1,25	1,25	7,5	10,1667
1,25	1,25	7,5	10,5999
1,25	1,25	7,5	11,103
1,25	1,25	7,5	10,0968
2	0,5	7,5	8,44776
0,5	2	7,5	11,0191
2	2	7,5	26,1818
0,5	0,5	7,5	5,45715
